# Targeting the activated allosteric conformation of the endothelin receptor B in melanoma with an antibody-drug conjugate: mechanisms and therapeutic efficacy

**DOI:** 10.1038/s44276-024-00109-y

**Published:** 2025-01-20

**Authors:** Amaury Herbet, Marie Hautière, Frédéric Jean-Alphonse, Delphine Vivier, Christophe Leboeuf, Narciso Costa, Aloïse Mabondzo, Guilhem Bousquet, Franck Denat, Eric Reiter, Didier Boquet

**Affiliations:** 1 Université Paris-Saclay, CEA, INRAE, Médicaments et Technologies pour la Santé (MTS), SPI, Laboratoire d’Etude de l’Unité Neurovasculaire et Innovation Thérapeutique (LENIT), Gif-sur-Yvette, France; 2 INRAE, CNRS, Université de Tours, PRC, Nouzilly, France; 3 Inria, Inria Saclay-Ile-de-France, Palaiseau, France; 4 Institut de Chimie Moléculaire de l’Université de Bourgogne, ICMUB UMR CNRS 6302, Université de Bourgogne, Dijon, France; 5 Université Paris Cité, INSERM, UMR_S942 MASCOT, Paris, France; 6 Université Sorbonne Paris Nord, Villetaneuse, France

## Abstract

**Background:**

Endothelin 1 receptors are one of the drivers of tumor progression in many cancers. Inhibition of their signaling pathways with antagonist drugs has been the subject of numerous clinical trials, but the results have not met expectations probably due to the high endothelin concentrations in the tumor microenvironment and their unusually high affinity for their receptors.

**Methods:**

We previously reported the rendomab B49 antibody (RB49) exhibiting a preferential affinity for the activated conformation of human endothelin B receptor (ET_B_), not displaced by high endothelin levels, and without any pharmacological properties that could inhibit the division of melanoma cells. In this context, we have developed xiRB49-MMAE, a chimeric antibody-drug conjugated (ADC) to monomethyl auristatin E. We have characterized its physicochemical properties, studied its binding mechanisms, and evaluated its therapeutic potential in a preclinical model. Immunohistochemical analysis of metastatic melanoma lymph nodes evaluated RB49 as a diagnostic tool for patient stratification.

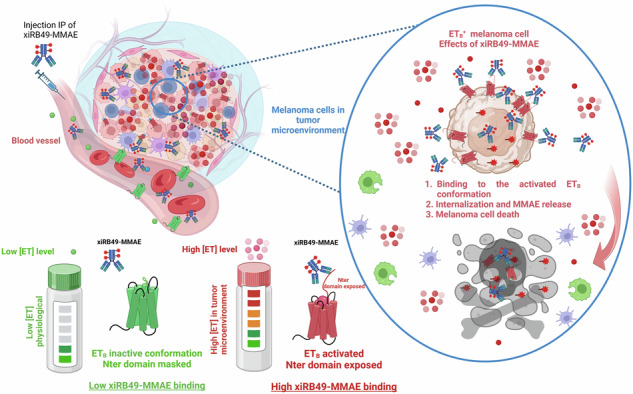

**Results:**

xiRB49-MMAE showed high efficacy against melanoma cells and ET_B_^+^ xenograft tumor models. IHC studies indicated that 100% of melanoma patient lymph node biopsies were RB49-positive.

**Conclusions:**

xiRB49-MMAE is a promising drug candidate for clinical trials in ET_B_^+^ tumors. RB49 could be used as a diagnostic tool for patient stratification.

## Background

With an estimated 325,000 new cases worldwide and 57,000 deaths from melanoma in 2020, this cancer is increasing dramatically. If current rates continue, they could increase by 68% by 2040 [1]. While immune checkpoint inhibitors (ICIs) have revolutionized the treatment of metastatic melanoma, ICI treatments are associated with immune-related adverse events that lead to treatment suspension in a substantial fraction of patients [2, 3]. New treatment options are needed for patients not eligible for ICIs, and ET_B_, a target already approved by the Food and Drug Administration, represents a promising option for melanoma treatment, as evidenced by numerous therapeutic trials targeting endothelin (ET) receptors [4–6]. ET axis dysregulation in cancer is associated with overexpression of ET_A_ and/or ET_B_ and their ligands ET 1 (ET-1), 2 (ET-2), and 3 (ET-3) [7–10]. ET_B_ is particularly overexpressed in melanoma cells and plays a crucial role in tumor progression and metastasis [11, 12]. For these reasons, we have developed several antibodies targeting ET_B_, including rendomab B1 (RB1), rendomab B4 (RB4), and rendomab B49 (RB49) [13–15]. Due to its remarkable affinity for a conformational recognition of ET_B_, RB49 was selected. Nevertheless, as it did not exhibit pharmacological properties that could inhibit the division of melanoma cells, we developed an ADC. With over 1,500 clinical trials and many ADCs approved or in late-phase clinical trials, this approach to cancer treatment is now mature [16]. We investigated the properties and therapeutic potential of xiRB49-MMAE to target and eliminate ET_B_^+^ melanoma in a preclinical model and evaluated the potential clinical utility of RB49 as a companion diagnostic tool for patient stratification.

## Methods

### RB49 chimerization

RB49 was produced from hybridoma and purified on protein A. For xiRB49 expression, the VH sequence was inserted into a pTT5 vector encoding the human IgG1, and the VL sequence into a pTT5 vector encoding the human kappa. Both vectors were transfected into ExpiCHO-S cells (Thermo Fisher Scientific (TFS), Waltham, Massachusetts, USA - Cat # A29127) and expression was induced following the manufacturer’s instructions. xiRB49 was purified using Protein A (GE Healthcare, Chicago, Illinois, USA - Cat # Cat.17-0402-03) and dialyzed in the final step against PBS buffer (TFS- Cat # 806552).

### Physicochemical properties

Hydrodynamic radius measurement was determined using Dynapro nanostar^®^ (Wyatt Technology, Santa Barbara, California, USA), The absorbance at 280 nm was used to calculate the antibody concentration (Spectramax M3^®^, Molecular Devices, San José, California, USA). The antibody stability measurement was determined using the Tycho NT.6 instrument (Nanotemper, München, Germany). It generated thermal unfolding profiles and identified the temperature inflection (Ti) as an indicator of changes in protein structural integrity. All experiments were performed in triplicate using a final antibody concentration of 0.5 mg/ml in PBS buffer at pH 7.4.

### xiRB49 MMAE conjugation

A solution of xiRB49 (1 mg/ml in PBS pH 7.4) was reacted with Tris (2-carboxyethyl) phosphine hydrochloride (TCEP; 10 mM, 10 equivalents) at 37 °C for 1 h. TCEP was removed using a centrifugal filter (Millipore Corp., Billerica, MA - Cat # UFC201024). Then, a solution of MaleimidoCaproyl-Valine-Citrulline-ParaAminoBenzyloxycarbonyl-Monomethyl Auristatin E, MC-VC-PAB-MMAE (10 mM, 15 equivalents), was added to the reduced antibody. The reaction was conducted at 37 °C for 30 min, followed by purification using a size exclusion column (Cytiva, Danaher Corporation Washington, USA - Cat # 17085101) and concentration with a centrifugal filter. For mass spectrometry analysis, a small sample of xiRB49-MMAE (20 µg) was deglycosylated using PNGaseF enzyme (NEB, Ipswich, MA - Cat # P0704L) at 37 °C for 1 h. The drug-to-antibody ratio (DAR) was estimated using denaturing mass spectrometry, where deglycosylated and reduced ADC samples were analyzed by reverse-phase HPLC coupled to a high-resolution Orbitrap mass spectrometer (TFS, Cat # Exploris 240) with an ESI source (positive mode). The number of cargos attached was determined by comparing native and conjugated heavy and light chains, resulting in an average DAR of 8. Aggregation of xiRB49-MMAE was evaluated by size exclusion chromatography (SEC) using an AdvanceBio SEC column (Agilent Technologies, Santa Clara, California, USA - Cat # PL1580-3301). The column was eluted at 25 °C with 200 mM ammonium acetate (pH 6.9) at a flow rate of 0.5 ml/min.

### Cell culture

All cell culture reagents were from TFS. Melanoma cell line UACC-257, from the DCTD Tumor Repository (Frederick, Maryland, USA), was cultured in RPMI-1640 (Cat # 12633012). All media were supplemented with 10% fetal calf serum (Cat # A4736401), 1 mM pyruvate, 1% nonessential amino acids, 2 mM glutamine, 100 U/ml penicillin, and 100 µg/ml streptomycin (Cat # 11530396, Cat # 11350912, Cat # 11539876, Cat # 11548876). HEK293A cells from TFS (Cat # R70507) were cultured in DMEM (Eurobio, Cat # CM1DME68.01) supplemented with 10% fetal calf serum (Eurobio, Cat # CVFSVF06-01), 100 U/ml penicillin and 100 µg/ml streptomycin (TFS, Cat # 5140122). For bioluminescence resonance energy transfer (BRET) assays, HEK293 cells were transfected with plasmids encoding BRET sensors using MetafectenePro (Biontex, Munich, GERMANY- Cat # T040-5.0). Cells were maintained at 37 °C in a humidified atmosphere of 5% CO_2_. All cells were mycoplasma-free.

### BRET assays

To determine changes in protein-protein interaction or in the activation of signaling biosensors, 50,000 cells/well in 96-well plates (Greiner, Les Ulis, FRANCE - Cat #655098) of HEK293 cells were seeded and transiently transfected using MetafectenePro with plasmids encoding Rluc8 as donor with plasmids encoding the acceptor, Venus or yPet. Before the experiment, cells were incubated with PBS with the Rluc8 substrate coelenterazine at 5 µM (Interchim, France - Cat #R3078C). Stimulations with ligands and antibodies were done in PBS/coelenterazine at 5 µM during the indicated time. Emission signals at 480 nm and 540 nm were acquired every 60 s in real-time using a Mithras 2 LB943 plate reader (Berthold). BRET signal changes were determined by analyzing the ratio 480/540 or 540/480.

### G protein recruitment

G protein coupling selectivity was determined by measuring the recruitment of the G protein BRET (bioluminescence resonance energy transfer) sensors (mini G proteins) by the endothelin receptors C-terminally fused with Rluc8 [17] upon ligand stimulation. Briefly, 50,000 HEK293 cells were seeded in 96 wells plate coated with poly-lysine and transiently transfected using metafectenePro (Biontex Laboratories GmbH - Cat# T040-1.0) for 48 hours with 50 ng of plasmid encoding for either ET_B_^Rluc8^ or ET_A_^Rluc8^ in combination with 30 ng mGi^Venus^, mGq^Venus^, mGs^Venus^ or mG12^Venus^. All plasmids were kindly provided by Prof. Nevin Lambert (Augusta University, USA). Following transfection, cells were washed once with PBS then pre-incubated with PBS containing 10 µM of the luciferase substrate coelenterazine-h (Interchim France - Cat # R3078K) for at least 5 minutes. Then, buffer was replaced with increasing concentrations of ET-3 (0.3 nM to 1000 nM), with or without 100 nM of RB49 or xiRB49, in PBS/coelenterazine buffer. Data acquisition was monitored at 37 °C every minute for 60 minutes using a plate reader (Mithras2, Berthold) using filters at 480 and 530 nm.

### cAMP signaling studies

cAMP signaling was determined by measuring signal dynamics in live cells by BRET. 48 h prior to the experiment, cells were transfected with 60 ng of plasmids encoding either ET_B_ or ET_A_ (in-house CEA) and 15 ng of plasmid encoding the cAMP biosensor ^Nluc^Epac^vv^ (gift from Prof Kirill Martemyanov, Scripp Institute) [18]. Cells were challenged with 5 µM forskolin to induce cAMP production in the presence or not of ET-3 at 1 to 30 nM and RB49 or xiRB49 at 100 nM. cAMP signaling dynamics were acquired every 60 s for at least 50 min.

### Flow cytometry analysis

To determine the apparent dissociation constant (Kd) binding experiments were performed. For flow cytometry analysis (FACS), cells were harvested at 90% confluence using a versene solution (PBS – 0.05% EDTA), washed once in PBS, and suspended in a saturation buffer (0.5% BSA, 5% normal goat serum NGS, D-PBS) at a concentration of 3.10^6^ cells/ml and incubated overnight at 4 °C with different concentrations of antibodies (0.005 to 100 nM). Cells were washed three times in cold PBS and suspended with 200 µl of saturation buffer containing either the secondary antibody anti-mouse Alexa Fluor™ 488 (AF488) (TFS - Cat # A10684) or anti-human AF488, (TFS - Cat # H10120) and incubated for 2 h at 4 °C. Cells, PBS washed three times, were suspended in 200 µl of FACS buffer (Thermo Fisher Scientific – FACSFlow Cat # 12756528) containing no fetal bovine serum (FBS), and the fluorescence intensity of 10,000 cells was quantified with a FACS Calibur (BD Bioscience, San Jose, CA, USA). For the competition experiments, cells were incubated with antibodies as previously described, with the addition of 300 nM of ETs (100 nm each of ET-1 (Sigma - Cat # E7764), ET-2 (Sigma - Cat #E9012) and ET-3, (Sigma - Cat # E9137) for each point in the range. Incubations were performed at 4 °C to limit receptor internalization during overnight incubation allowing equilibrium to be reached.

The data were analyzed using Prism 9.4 (GraphPad Software San Diego, CA, USA) to determine the Kd and the maximum specific binding value (Bmax) using a non-linear regression fit: site-specific linkage after checking that we had a normal distribution of these data by a D’Agostino & Pearson test. The results are expressed as percent mean fluorescence intensity (MFI) after normalizing to a high and a low value. Statistical significance between two groups of unpaired data was assessed by a parametric t-test. *P* value symbol: ns *P* > 0.05, *P ≤ 0.05, **P ≤ 0.01, ***P ≤ 0.001 and ****P ≤ 0.0001.

### Internalization studies

Cells were seeded on glass coverslips at a density of 100,000 cells per cm^2^ and incubated with mAb (5 nM) for 2 h at 4 °C for membrane labeling. After three PBS washes, secondary mAb AF488 were incubated either for 2 h at 4 °C for membrane labeling or for 1 h at 37 °C to observe mAb-ET_B_ complex internalization. After three PBS washes, one drop of ProLong Gold Antifade Mountant (TFS, Cat # P10144) was deposited on each slide and a cover glass slide was applied. Observations were performed at 40-fold magnification with the Apotome fluorescent microscope (ZEISS, Oberkochen, Germany).

### Endocytosis quantification by BRET

Receptor endocytosis was determined by measuring the localization of the receptor at the plasma membrane by bystander BRET. HEK293 cells were transfected with 50 ng of ET_B_-Rluc8 20 ng of and 20 ng of yPet-Lyn. 48 h after transfection, cells were incubated in PBS coelenterazine (5 µM) and then stimulated with ET-3 (100 nM) with or without RB49 or xiRB49 at 100 nM for at least 60 min. BRET signals were acquired every 60 s. A decreased BRET ratio (emission 480/540) relates to the disappearance of the ET_B_ at the plasma membrane due to its internalization.

### Cytotoxic assay

Cell cytotoxicity was monitored using a specific assay (Abcam, Cambridge, UK- Cat # 112118). This cytotoxicity assay is based on the measurement of mitochondrial dehydrogenase activity using a specific dye. This assay evaluates the metabolic activity of viable cells. Cells were plated at 1500 cells/well in 100 µl culture medium. The following day, 100 µl of increasing concentrations of mAb, diluted in culture medium, were added to each well. Cells were incubated for 96 h at 37 °C in a 5% CO_2_ incubator. The OD570/OD605 nm ratio was used to determine cell viability in each well in triplicate according to the instructions. Data were analyzed using Prism 9.4 (GraphPad Software San Diego, CA, USA) to determine the half maximal inhibitory concentration (IC_50_) using the variable slope model after checking that we had a normal distribution of these data by a D’Agostino & Pearson test.

### Immunohistochemistry

Endothelin receptor B expression was assessed in 10 melanoma metastatic lymph nodes obtained from 10 different patients treated at Saint-Louis Hospital France, before 2015. They had been previously included in a published biomarker study (doi: 10.1002/ctm2.198) approved by the Clinical-Research-Board-Ethics-Committee (CPP-Ile-de-France#13218). The 10 biopsies analyzed here have been randomly selected among a cohort of 101 patients diagnosed between 2009 and 2014 with a regional macroscopic lymph node metastatic melanoma and without distant metastases at the time of initial surgery. Immunostaining was evaluated by two specialists with expertise in experimental pathology Prof. Anne Janin and Prof. Guilhem Bousquet. Immunohistochemical analyses were carried out on 5 μm paraffin sections with an indirect immunoperoxidase method, using mouse anti-human antibody RB49 directed against ET_B_ and a DABMap detection kit (Roche Diagnostics, Meylan, France). Systematic controls with an irrelevant primary antibody of the same isotype were assessed. A CHO cell line overexpressing ET_B_ was used as positive control, and normal liver and skin as negative controls. Tissue sections were analyzed using an Olympus AX 70 microscope, with a 0.344 mm^2^ field size at X400 magnification (Olympus, Tokyo, Japan).

### In vivo experiments

Experiments were realized according to the ARRIVE Guidelines 2.0. Female athymic nude mice (10 weeks old) from Janvier Labs (Le Genest-Saint-Isle, France) were housed in a specific pathogen-free animal facility, according to French animal experimentation regulations and ethical principles. The experiments were started after at least 5 days of acclimatization of the animals. An analysis using G-Power 3.1 statistical software was used to determine the number of animals required for the study. Using a two-tailed t-test to compare two independent means, with an effect size of 2, a significance level of 5%, and a statistical power of 95%, the software recommends a total of 8 animals per group. To establish subcutaneous xenografts, UACC-257 cells (5.10^6^ in 100 µl) were injected into anesthetized mice (3% isoflurane). Tumor size measurements were taken after approximately one month when the tumors reached approximately 100 mm^3^, and the therapeutic protocol was initiated. Animals with no growth or low tumor growth were excluded from the study. The mice were randomized (randomization assignment) into four groups, each consisting of eight mice: i) control vehicle group 1, ii) control group 2 treated with naked xiRB49 at 4 mg/kg, iii) group 3 treated with xiRB49-MMAE at 1 mg/kg, and iv) group 4 treated with xiRB49-MMAE at 4 mg/kg. On Day 47 and Day 80, all groups received intraperitoneal injections. Treatments were randomly assigned to different subjects to ensure that each treatment had an equal chance of being administered first, thereby minimizing order effects but no blinding was done. Tumor sizes were measured twice a week using calipers. The greatest longitudinal diameter (L) and the greatest transverse diameter (W) of the tumor were recorded, and the tumor volume (V) was calculated using the formula: V = ½ (L × W^2^). Normal distribution was observed for control groups not for treated groups with a coefficient of variation similar closed to 60% for all groups.

### Statistics

AEC50 values were calculated by performing nonlinear regression analysis on concentration-response curves. For *in vivo* experiments, statistical analysis was conducted using two-way ANOVA with post-hoc Bonferroni correction for TGImax, and the Mantel-Cox log-rank test for TGD using GraphPad Prism software (GraphPad Software, La Jolla, CA).

## Results

### xiRB49 retained the same conformational binding properties as RB49

#### A nanomolar affinity for ET_B_ and a fixation not abolished by endothelins

**Fig. 1:**
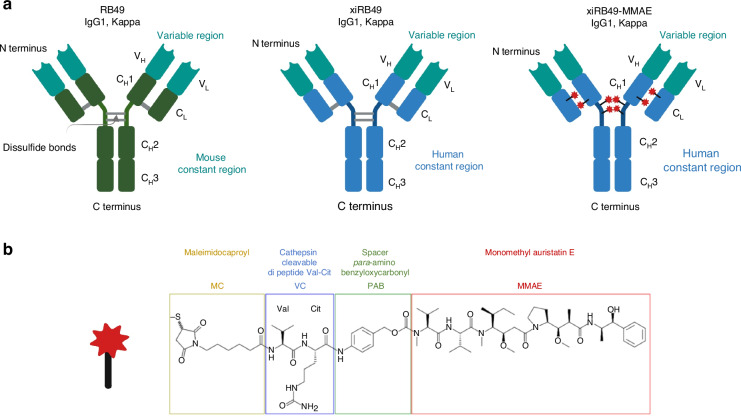
Schematic representations and structural insights into RB49-based antibodies and linker MMAE. **a** Schematic representations of RB49, xiRB49, and xiRB49-MMAE antibodies. **b** Structure of linker MMAE.

**Fig. 2:**
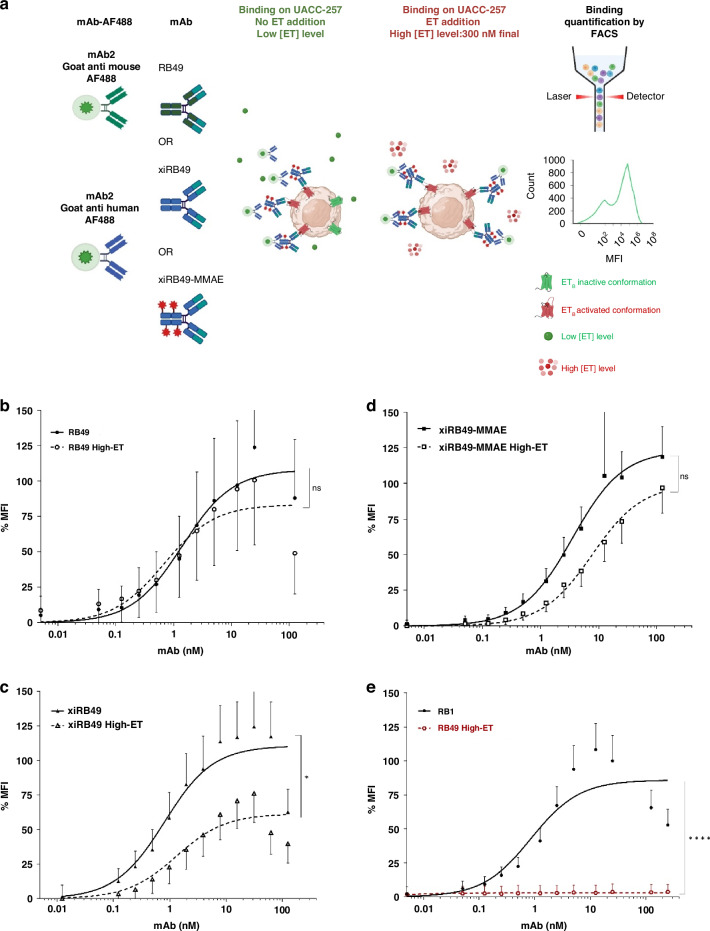
Binding profiles of RB49, xiRB49, xiRB49-MMAE, and RB1 on ET_B_^+^ human melanoma UACC-257 cells with or without high endothelin levels. **a** Schematic representation of mAb binding experiments with or without ET on ET_B_^+^ human melanoma UACC-257 cells. Binding curves were generated by plotting normalized MFI (%MFI) versus mAb concentration without or with high concentrations of ET, ET-1, ET-2, and ET-3 at 100 nM each. **b** RB49 binding curves, without ET, black dot, and black solid line, or with high ET concentrations, empty circles, and black dotted line. No significant difference was observed between the two conditions. **c** xiRB49 binding curves without ET, black triangle, and solid black line, or with high ET concentrations, empty triangle, and black dotted line. xiRB49 binding is maintained with a decrease. **d** xiRB49-MMAE binding curves without ET, black square, and solid black line, or with high ET concentrations, empty square, and black dotted line. No significant difference was observed between the two conditions. **e** RB1 binding curve without ET, black dot, and solid black line or with high ET concentrations, empty red dot, and red dotted line. RB1 binding is completely abolished at high ET concentrations.

The RB49 mAb was chimerized with human IgG1 and Kappa isotypes, resulting in xiRB49 (Fig. 1a) [19]. RB49 and xiRB49 demonstrate selectivity for ET_B_, showing no binding towards ET_A_ [15]. To assess whether the binding characteristics of RB49 were maintained after chimerization, we performed a series of binding experiments by cytometry on ET_B_^+^ UACC-257 cells with or without the addition of endothelins (ET) at a final concentration of 300 nM (Fig. 2a). We chose an extra physiological concentration with a mixture of three ETs (100 nM) to be in a very drastic condition since the plasma concentration is classically observed at 5 pM with a peak at 0.5 nM [7]. As observed in Fig. 2b, the binding curves of RB49 (black curve) and those with high ET concentrations (dotted line) were comparable. The Kd and Bmax, for RB49 were found to be Kd = 1.4 ± 0.02 nM and Bmax = 108.1 ± 0.5, which were similar to the values obtained for RB49 in the presence of a high concentration of all three ETs (High ET: 300 nM final): Kd = 0.7 ± 0.02 nM and Bmax = 83.3 ± 0.4 (see Table s1 in the supplementary informations SI). In Fig. 2c, we determined the binding values for xiRB49 (black curve) and xiRB49 with high ET concentrations (dotted line), yielding Kd = 0.8 ± 0.01 nM and Bmax = 110.7 ± 0.2, and Kd = 1.3 ± 0.02 nM and Bmax = 61.5 ± 0.2, respectively (see Table s2 in SI). These results confirmed that xiRB49 maintained its nanomolar affinity and, importantly, its binding to ET_B_, even in the presence of high concentrations of ETs. The significant excess of ETs (300 nM final) may explain the reduction in Bmax observed for RB49, xiRB49, and xiRB49-MMAE, see below, at high ET due to a decrease of ET_B_ at the cell membrane. We further examined the impact of individual ET on RB49 and xiRB49 binding. The individual competition binding assays of RB49 and xiRB49 with 100 nM ET-1, ET-2, or ET-3 are presented in SI Fig. s1 and s2, and in Tables s1, s2. Neither RB49 nor xiRB49 demonstrated any substantial effect on affinity or Bmax in the presence of these ETs. Equivalent results were observed for xiRB49-MMAE with or without high ET concentration. In Fig. 2d, we determined the binding values for xiRB49-MMAE (black curve) and xiRB49-MMAE with high ET concentrations (dotted line), yielding Kd = 3.5 ± 0.05 nM and Bmax = 123.2 ± 0.5, and Kd = 7.7 ± 0.15 nM and Bmax = 99.7 ± 0.5, respectively (see Table s3 in the SI). The competition binding assays of xiRB49-MMAE with 100 nM ET-1, ET-2, or ET-3 are presented in SI Fig. s3 and Table s3. It is worth noting that this lack of competition was not observed with all our antibodies such as Rendomab B1 (RB1). RB1 high-affinity binding (Kd = 0.9 ± 0.02 nM) was completely abolished in the presence of high ET levels (Fig. 2e and in the SI, Schema a, and Table s4), as previously described [13]. In conclusion, xiRB49 retains the properties of RB49 in terms of affinity (nM), specificity, and absence of binding to CHO-WT cells, and its binding is not abolished even in the presence of high ET concentrations. Conjugation of MMAE to xiRB49 did not alter these properties.

#### RB49 or xiRB49 pharmacological and internalization studies

**Fig. 3:**
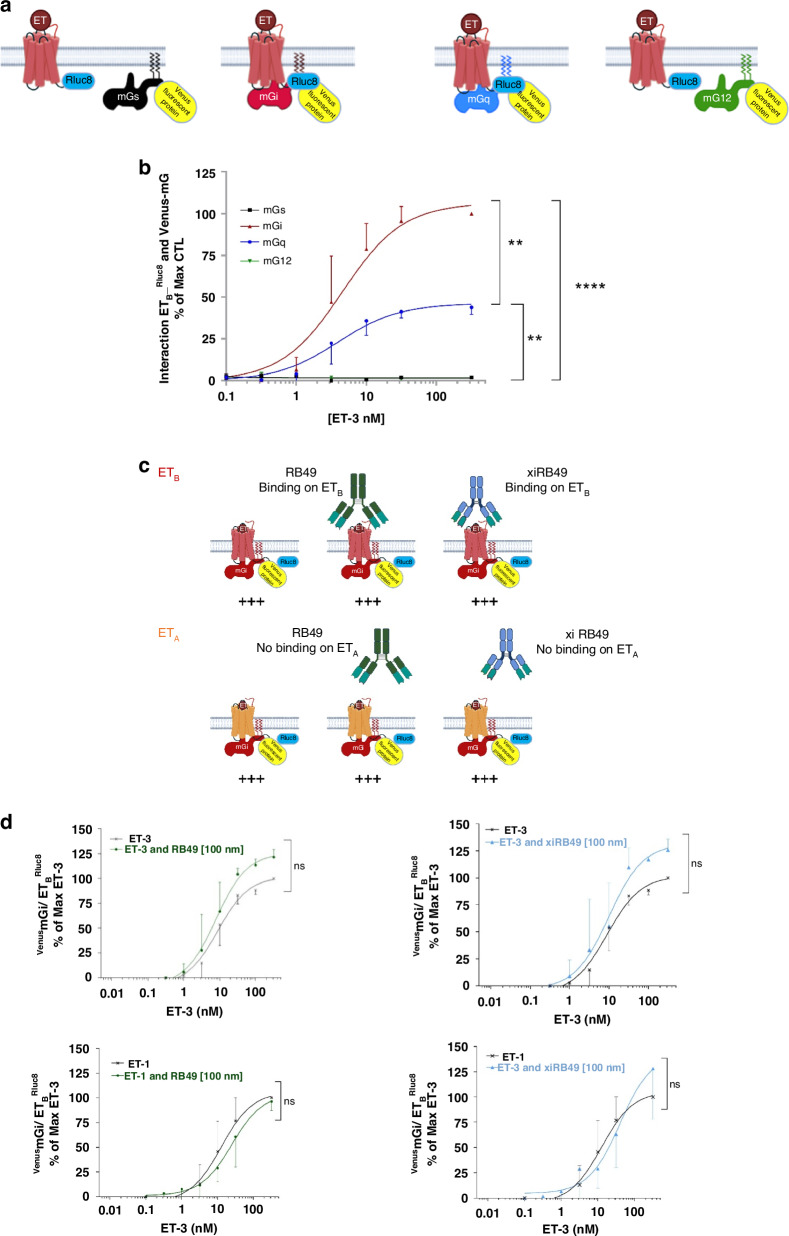
Bret biosensor analysis of ET_A_ and ET_B_ receptor coupling and the impact of RB49 and xiRB49 on miniG proteins binding. **a** Schematic representation of the BRET biosensor miniG proteins (mG) fused to venus and ET receptor-Rluc8. **b** Dose-responses curves of ET-3 stimulation of ET_B_ show the preferential coupling of ET_B_ with Gi (red) over Gq (blue) and no coupling with Gs (black) and G12 (green). **c** Schematic representation of mGi coupling to ET_B_ or ET_A_ in the presence of ET with or without RB49 or xiRB49. **d** Dose-response of ET-3 or ET-1 on mGi binding to ET_B_ (top) or ET_A_ (bottom) in the presence or absence of 100 nM of RB49 (green) or xiRB49 (blue). No modification was observed.

**Fig. 4:**
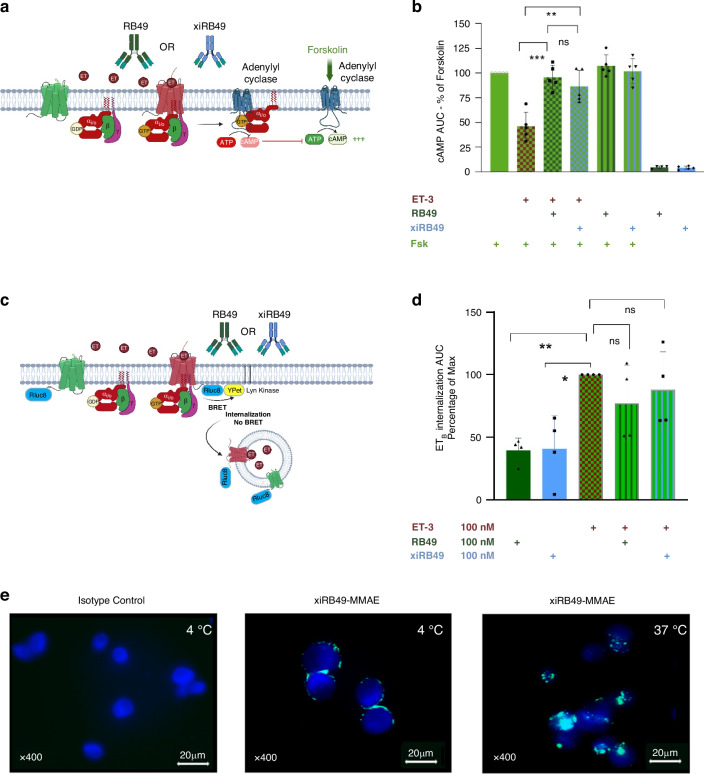
Impact of endothelin, RB49, and xiRB49 on cAMP production. xiRB49-MMAE internalization in ET_B_^+^ human melanoma UACC-257 cells. **a** Schematic representation of the inhibition of forskolin-induced adenylyl cyclase-dependent production of cAMP by activation of Gi by ET on ET_B_. **b** cAMP production is inhibited by ET-3. This ET-dependent cAMP inhibition is completely reversed in the presence of ET-3 at 3 nM and RB49 or xiRB49 at 100 nM. No forskolin-induced cAMP change is observed in the presence of RB49 or xiRB49 alone. No cAMP is induced by the addition of RB49 or xiRB49 alone without forskolin. **c** Schematic representation of ET_B_ endocytosis measurement by bystander BRET using ET_B_-Rluc8 and the membrane-attached sensor Lyn-Ypet. Upon endothelin stimulation with or without mAb, receptor endocytosis into the endosome results in a decrease of the BRET signal, ET-3 alone, 100% internalization. **d** RB49 (green) and xiRB49 (blue) at 100 nM induced low ET_B_ endocytosis compared to ET3 alone and the addition of RB49 or xiRB49 does not alter endothelin-mediated endocytosis. **e** Apotome experiment (Isotype control) UACC-257 cells with isotype control. Blue staining of cell nuclei is observed, and no signal of fluorescence is detected. (xiRB49-MMAE-4 °C) UACC-257 cells were stained with 10 nM of xiRB49-MMAE at 4 °C and revealed with a secondary antibody AF488. The picture is merged with blue staining. Green labeling of the membrane is observed. (xiRB49-MMAE-37 °C) UACC-257 cells were stained with 10 nM of xiRB49-MMAE and revealed with a secondary antibody AF488. Incubation is done at 37 °C. The picture is merged with blue staining. We observed a cytoplasmic fluorescence witnessing internalization of xiRB49-MMAE.

We investigated the effects of RB49 or xiRB49 binding to activated ET_B_ on G-protein coupling using BRET. For this purpose, we studied its coupling to the four subfamilies of heterotrimeric G protein sensors for G*α*q/11, G*α*s, G*α*i/o, and G*α*12/13, namely the miniG proteins mGs, mGi, mGq, and mG12. MiniG proteins coupled to the fluorescent protein venus were co-transfected in HEK293 cells with ET_B_ fused in its C-tail with the bioluminescent enzyme Renilla luciferase Rluc8 (ET_B_-Rluc8 (Fig. 3a) [17]. We added ET-3 because it is the ET_B_-specific ET and no ET_A_-binding activity was found in the melanoma microenvironment associated with EDNRB overexpression (see dataset a1, a2 in SI) [12, 20]. After adding ET-3, ET_B_ preferentially recruited mGi and secondarily mGq, no detectable mGs and mG12 association was observed (Fig. 3b). We then investigated the effects of RB49 or xiRB49 binding on mGi-ET_B_ coupling induced by ET-3 (Fig. 3c, d), no significant modification was detected in Gi recruitment to ET_B_ in the presence of RB49 or xiRB49. A similar result was observed on ET-1-induced mGi recruitment to ET_A_ knowing that no ET_A_ binding of these mAbs was observed. We also assessed the effects of RB49 or xiRB49 binding on the inhibition of adenyl cyclase (AC) by G*α*i/o protein after ET_B_ activation by ET-3 (Fig. 4a). We directly activated AC with 5 µM forskolin and then measured the cAMP in live cells using the BRET sensor [18]. In these conditions, the area under the curve measured was our 100% of cAMP production green bar (Fig. 4b). We studied the effect of ET-3 on forskolin-induced cAMP production (Fig. 4b and Fig. s4 ET_B_ in SI). For all concentrations of ET-3 (1–10 nM), forskolin-induced cAMP production was decreased by 50% on average, which is consistent with Gi-mediated AC inhibition. Co-incubation of either RB49 or xiRB49 with ET-3 reversed the antagonistic effect of ET-3 to nearly 100% of cAMP production, *suggesting a conformational ET*_*B*_ *modification induced by mAb binding that prevented the activation of recruited Gαi/o without altering its coupling* (Fig. 3d). As expected, no reverse effect of RB49 or xiRB49 on ET_A_ was observed (Fig. s4 ET_A_ in SI). No effect was observed on forskolin-induced cAMP production when RB49 and xiRB49 were added to ET_B_ *without* ET-3. Similarly, when RB49 or xiRB49 were added alone to ET_B_, no effect was observed on cAMP production. *The last two experiments support the hypothesis that RB49 or xiRB49 highly modulates ET*_*B*_ *activity only when a ligand, in this case ET-3, is bound*.

We studied the internalization of ET_B_ by BRET by measuring the localization at the plasma membrane of ET_B_-Rluc8 using the plasma localized sensor YPet-Lyn (Fig. 4c, d). ET-3 stimulation (shown in green with squared red), corresponding to our 100% internalization, indicated that ET_B_ was well internalized, as expected (Fig. 4d). In the absence of ET-3, but with RB49 or xiRB49 alone, we observed a basal internalization of approximately 40%, which may reflect the equilibrium between different conformational states of ET_B_ recognized/unrecognized by the antibodies. However, in presence of ET-3 and RB49 or xiRB49, we observed an increase of ET_B_ internalization, *coherent with the hypothesis that RB49 or xiRB49 highly modulates ET*_*B*_ *activity only when a ligand is bound*, thereby validating an ADC therapeutic approach. We also observed the internalization of RB49 or xiRB49-bound to ET_B_ by an immunofluorescence approach using the melanoma cell line UACC-257 (Fig. s5a and s5b in SI). As the addition of xiRB49 to tumor cells had no effect on their growth (see below), we decided to conjugate it with the cytotoxic monomethyl auristatin E (xiRB49-MMAE) (Fig. 1a, b).

### xiRB49-MMAE displays potent anti-melanoma activity

#### xiRB49-MMAE characterization

After reduction of the disulfide bonds, maleimide-MMAE was site-specifically conjugated to xiRB49. A DAR of 8 – one payload per light chain and three per heavy chain – was estimated using mass spectrometry in denaturing conditions, indicating that all cysteines were coupled with MMAE (Fig. s6 a in SI). No aggregation was detected by SEC analysis or by DLS (Fig. s6b, c in SI). The Rh values of 4.6 ± 0.2 nm and 6.8 ± 0.1 nm, respectively for xiRB49 and xiRB49-MMAE, showed a moderate increase due to the addition of 8 drugs (Table s5 in SI). Thermal denaturation experiments were conducted with native and conjugated xiRB49 to compare inflection temperatures reflecting changes in either constant (Ti#1) or Fab (Ti#2) domains of the antibody (Fig. s6d in SI). Similar Ti#2 values were measured (89.4 °C ± 0.13 °C and 88.5 ± 0.17 °C respectively for xiRB49 and xiRB49-MMAE), while no Ti#1 was determined for the conjugated construct (Ti#1 was 74.1 ± 0.08 °C for native xiRB49) (Table s6 in SI). These results suggest destabilization of the constant domain following MMAE conjugation, but without alteration of its binding properties and affinity for ET_B_, as confirmed by flow cytometry experiments on ET_B_^+^ cells (Fig. 2d).

#### xiRB49-MMAE internalization and in vitro cytotoxicity study

As expected, internalization of xiRB49-MMAE in UACC-257 cells was observed by immunofluorescence, similarly to RB49 and xiRB49 (Fig. 4e). Thus, we proceeded with the cytotoxicity evaluation using a viability cell assay. Briefly, increasing concentrations of xiRB49-MMAE were incubated with melanoma cells before measuring cell viability. Zeocin™, a potent cytotoxic, was used as a positive control. With an IC_50_ of 0.4 ± 7.2 nM, the ADC was 65 times more effective than Zeocin (IC_50_ 27.1 ± 7.2 nM) in eradicating melanoma cells. Conversely, native xiRB49 did not display any toxicity (Fig. s8 and Table s7 in SI).

#### xiRB49-MMAE displays potent anti-melanoma activity in vivo

**Fig. 5:**
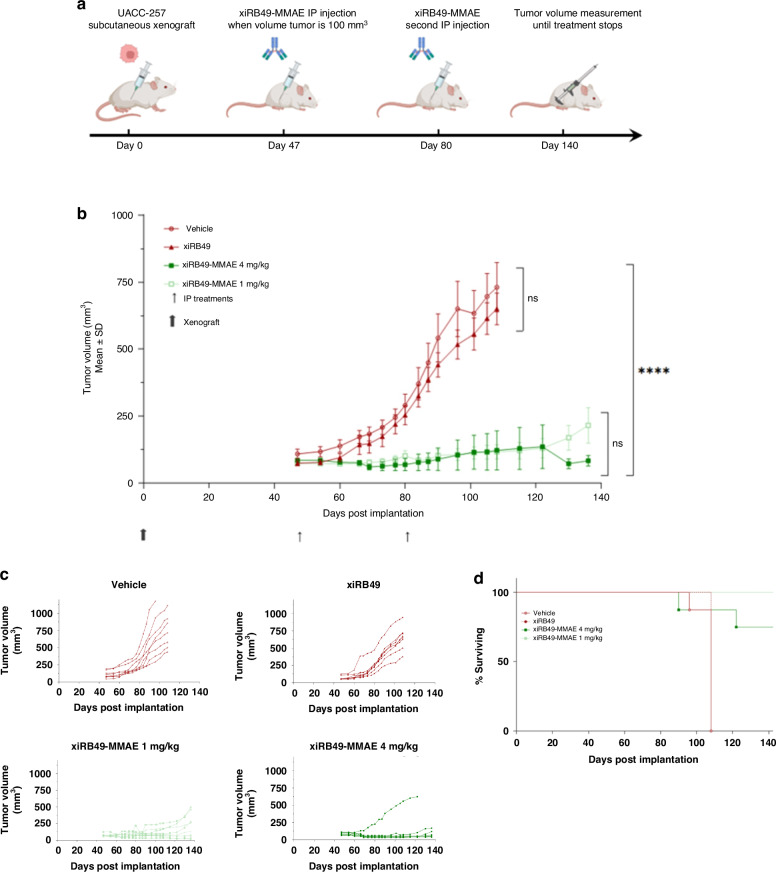
In vivo evaluation of xiRB49-MMAE antitumor activity and long-term survival study preclinical mouse models xenografted with ET_B_^+^ human melanoma UACC-257 cells. **a** Schematic representation of the in vivo experiments. Xenografts were performed on day 0, and mice were injected on days 47 and 80 post-xenograft. **b** Antitumor activity of xiRB49-MMAE is evaluated by tumor growth curves. Values represent the mean ± SEM derived from groups of 8 animals. The large black arrow indicates the day of xenografting and the light dark arrow indicates the day of xiRB49-MMAE administration. All treatment groups were significantly different from the control group (*p* < 0.0001) as determined by the Mann-Whitney test (*n* = 8 mice/group). **c** Antitumor activity of each animal per group. All mice in the Vehicle and xiRB49 groups were sacrificed on day 108 for ethical reasons. **d** xiRB49-MMAE administrations promote long-term survival. Image depicting survival in mice (time to tumor endpoint or death) compared to untreated controls.

Animals were randomized into four groups of 8, with each group receiving two intraperitoneal injections of the vehicle or xiRB49 (4 mg/kg) or xiRB49-MMAE (1 mg/kg or 4 mg/kg) at days 47 and 80 (Fig. 5a). The UACC-257 xenograft tumors were highly responsive to xiRB49-MMAE, resulting in dramatic inhibition of tumor progression during the 136 days (D136) of the study period (Fig. 5b). Both ADC-treated groups were significantly different from the control group (*p* < 0.0001) as determined by Mann-Whitney test (*n* = 8 mice/group). Conversely, vehicle or xiRB49 treatments exhibited no effect on tumor progression. All mice in the Vehicle and xiRB49 groups were sacrificed on day 108 for ethical reasons (due to significant ulceration or excessively large tumor volume). None of the animals treated with ADC, even at the highest dose (4 mg/kg), exhibited behavioral signs related to treatment toxicity and no loss of weight were observed. At D136, mice receiving 4 mg/kg of xiRB49-MMAE displayed no tumor progression (tumor size less than or equal to 100 mm^3^), compared to 50% for the group receiving 1 mg/kg of xiRB49-MMAE. For the 4 mg/kg dose, one mouse exhibited no response to treatment and one mouse was found dead in its cage overnight with a tumor volume of 55 mm^3^ without prior signs of disease or suffering Fig. 5c. To avoid an artificial decrease in tumor volume due to the death of this mouse, we decided to retain the last observation for this mouse up to day 140, even if the mouse had died before that date. This method helps prevent a misleading effect of the curve for the 4 mg/kg group. In terms of survival (Fig. 5d), 100% of the mice treated with 1 mg/kg were alive at the end of the study *versus* 75% for the mice treated with 4 mg/kg. The results might be underestimated due to the premature death of one mouse (day 90, tumor size 55 mm^3^) which showed no apparent clinical signs related to the implanted tumor or ADC injection. These *in vivo* results highlight the efficacy of xiRB49-MMAE in treatment of ET_B_^+^ melanoma, with no notable side effects on the mice.

#### IHC approach for patient stratification

**Fig. 6:**
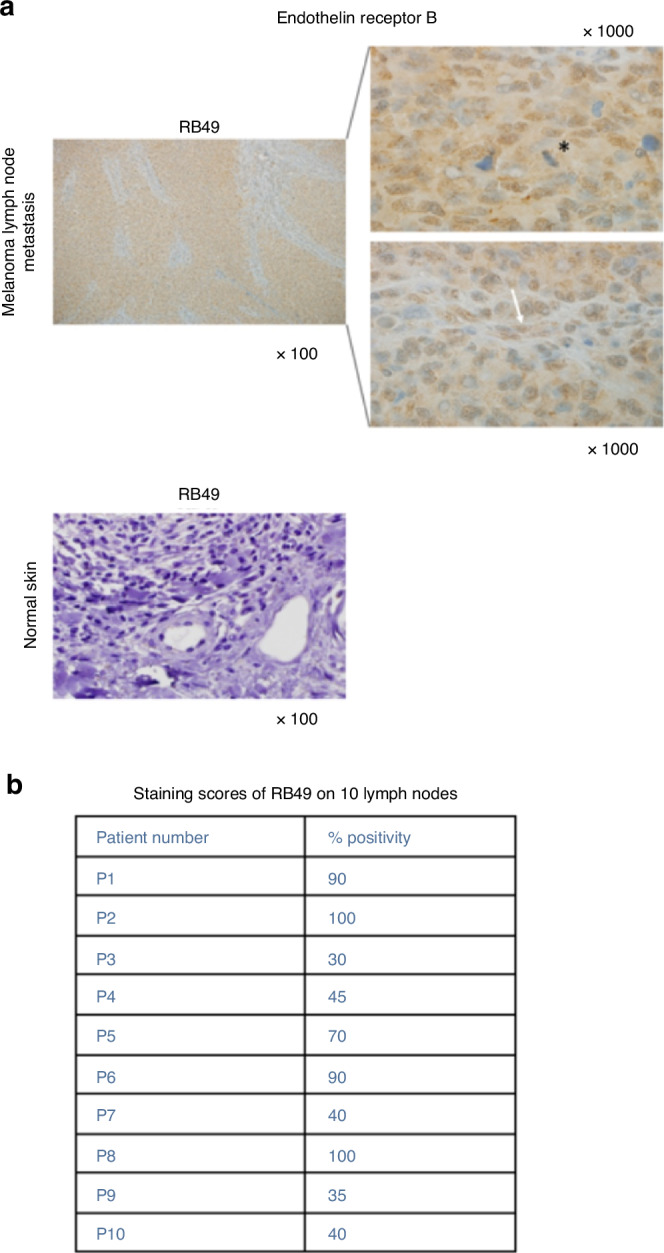
Immunohistochemistry study with RB49 of 10 lymph nodes from patients with melanoma (n = 10). **a** The figure is representative of the observations noted in the 10 biopsies. Intense and complete staining of the lymph nodes with RB49 was observed. ET_B_ was expressed in both melanoma cancer cells (black star) and tumor endothelial cells (white arrow). No staining was observed in normal skin, including normal blood vessels. **b** Staining score of RB49 on 10 lymph nodes.

In order to know if RB49 could be used as a companion test to stratify ET_B_^+^ melanoma patients eligible for possible treatment with xiRB49-MMAE, we performed an exploratory IHC screening study. Ten melanoma metastatic lymph nodes were analyzed after IHC treatment. All biopsies were positive with RB49 immunostaining. A representative result is shown in Fig. 6a. Intense and complete staining of the lymph node with RB49 was observed. ET_B_ was expressed both in melanoma cancer cells (black star) and tumor endothelial cells (white arrow). Staining scores of RB49 on 10 lymph nodes are presented in Fig. 6b. No staining was observed on normal skin or liver, including normal vessels. *This is additional evidence for recognition by RB49 of a particular conformation of the ET*_*B*_ *receptor expressed at the melanoma cell surface*. This first screening validates the possibility of studying whether RB49 can be used as a companion test to stratify patients eligible or not for future xiRB49-MMAE treatment.

## Discussion

The ET axis plays a significant role in various cancers, and by activating human ET_B_ through ET leads to processes like cell division, survival, angiogenesis, invasion, and metastasis [21]. Some tumor cells can initiate an autocrine loop by releasing significantly high levels of ET within the tumor microenvironment [22], which is a challenge for the use of antagonists in a therapeutic approach. We previously described RB49, which displayed high affinity and specificity for ET_B_ expressed on the UACC cell membrane [15]. In contrast to RB1, the binding of RB49 was not affected by the presence of ET. This remarkable property makes RB49 an attractive therapeutic antibody for targeting ET_B_^+^ tumor cells secreting high levels of ET. We have shown that xiRB49 retains all of its initial binding properties and confirmed its internalization in melanoma cells.

We investigated whether xiRB49 have intrinsic pharmacological properties. Because ET-3 plays an essential role in melanocyte development and the invasive behavior of melanoma cells, we examined G-protein coupling to ET_B_ after ET-3 binding with or without xiRB49 [23–25]. Having confirmed that Gαi/o was the main G-protein mobilized in our heterologous cellular model, we have shown that xiRB49 binding did not negatively affect the coupling of Gi. Nevertheless, xiRB49 binding to ET_B_ can reverse the AC inhibition induced by Gαi/o after ET-3 binding to ET_B_ suggesting that its binding to ET_B_ modifies its conformation and prevents the inhibition of AC by the Gαi/o protein normally induced by ET-3. Due to the lack of major pharmacological effects and cytotoxicity on tumor cells, it was decided to conjugate the xiRB49 with MMAE and to investigate its therapeutic potential for the treatment of melanoma [26, 27]. xiRB49-MMAE, with a DAR of 8, conserves its binding properties for ET_B_. The results of the *in vitro* and *in vivo* experiments highlight the anti-melanoma efficacy of xiRB49-MMAE. In our preclinical model, ADC treatment led to a dramatic inhibition of tumor progression over a 136-day study period. Importantly, no signs of toxicity were observed in the treated mice, even at the highest dose of 4 mg/kg. As expected, treatment with the vehicle or native xiRB49 had no significant effect on tumor regression, thus underscoring the importance of the ADC payload, MMAE, in conferring potent anti-melanoma activity on xiRB49 [28, 29]. After analyzing tumor volumes not exceeding 100 mm^3^, mice receiving 4 mg/kg of xiRB49-MMAE showed a higher proportion of no tumor progression (90%) compared to the group receiving 1 mg/kg (50%). Although the differences did not reach statistical significance, this observation suggests that higher doses of ADC may be more effective or that more repeated injections may be required to achieve complete tumor eradication. The survival analysis yielded promising results, with 100% of mice treated with 1 mg/kg of xiRB49-MMAE surviving until the end of the study. Although the survival rate in the 4 mg/kg group was slightly lower (75%), it is important to note that one mouse did not respond to the treatment, and another one died with no signs of deterioration in health. This variability in individual responses may be due to various factors, such as tumor heterogeneity and microenvironment, or the level of expression of the target biomarker, in our case ET_B_. Indeed, during tumor cell progression, ET_B_ expression may vary, allowing tumor cells to escape the action of ADC. For clinical application, it is therefore essential to have an antibody-based isotope imaging tracer to perform immuno-PET imaging that allows patient stratification. We are developing this new approach with xiRB49, as quantitative imaging would allow us to monitor the presence of ET_B_^+^ tumors [30, 31]. IHC of biopsy specimens is a classical approach for tumor characterization. We evaluated the potential use of RB49 as a companion test to stratify ET_B_^+^ melanoma patients eligible for xiRB49-MMAE treatment. Intense and complete staining with RB49 was observed with all melanoma metastatic lymph nodes. Notably, ET_B_ expression was observed in both melanoma cancer cells and tumor endothelial cells. Importantly, no staining was observed in normal skin, liver, and blood vessels. To explain these results, we hypothesize that when melanoma cells are present in the lymph nodes of patients the environment becomes very rich in ETs, thus switching all ET_B_ into an activated state recognized by RB49 and generating intense labeling. In the vessels, composed of endothelial cells expressing ET_B_, ET is transiently released and rapidly captured by ET_B_ which is rapidly internalized, making it poorly accessible to RB49. To ensure the efficacy of the treatment, future studies would need to evaluate RB49 staining on other metastatic sites on patient necropsies for example.

As with most G protein-coupled receptors, ligand binding to its orthosteric site induces conformational changes in ET_B_ and its published crystallographic structure revealed a mechanism by which ET binding causes an important rearrangement of the N-terminal domain, exposing it to the extracellular environment (Fig. s7) [32, 33]. We have previously shown that the RB49 epitope is located in this N-terminal domain and that RB49 ET_B_ binding is not affected by these ET-mediated conformational changes [15]. We also determined the persistence of RB49 binding in the presence of BQ788, a specific and potent antagonist of ET_B_ (data not shown).

Our results demonstrate that RB49, as well as xiRB49 and xiRB49-MMAE, preferentially binds to the ligand-activated ET_B_ conformation and that the latter is particularly overrepresented in tumors with an activated ET axis such as melanoma and underrepresented in normal tissues, limiting the off-targeting of xiRB49-MMAE. However, it is important to consider the differences in affinity of xiRB49 for ETB between mouse and human. xiRB49 binds to the human ET_B_ with high affinity and not the murin ET_B_. Therefore, the lack of toxicity observed in a mouse model does not necessarily predict the potential for toxicity in humans. Nevertheless, previous clinical trials targeting ET_B_ with an ADC (DEDN6552A MMAE conjugated) did not show high toxicity and allowed patients to continue receiving DEDN6552A for up to 17 cycles (1 year) [6]. These preclinical results strongly support the promising therapeutic potential of xiRB49-MMAE in precision medicine therapies for ET_B_^+^-expressing cancers and provide a compelling rationale for further clinical development of theranostic approaches.

## Supplementary information

Supplementary Information

## Data Availability

Data from this study can be found in the article and SI files. Additional data are available from the corresponding author, upon reasonable request. Materials are available only in the form of a collaboration agreement or MTA.
