# Supplementary material for: Targeting the activated allosteric conformation of the endothelin receptor B in melanoma with an antibody-drug conjugate: mechanisms and therapeutic efficacy
Source: BJC Rep. 2025 Jan 20;3:3. doi: 10.1038/s44276-024-00109-y (PMC11747117; doi:10.1038/s44276-024-00109-y)
Supplement: Supplementary file 1 — Supplementary Information [file 44276_2024_109_MOESM1_ESM.pdf]

Supplementary Information

Data set a1: Expression profiling array of endothelin receptor B in cutaneous malignant melanoma

EDNRB - Cutaneous malignant melanoma
Annotation: EDNRB, endothelin receptor type B
Organism: Homo sapiens
Reporter: GPL96, 204273_at (ID_REF), GDS1375, 1910 (Gene ID), **NM_000115**
DataSet type: Expression profiling by array, count, 70 samples
ID: 13263100

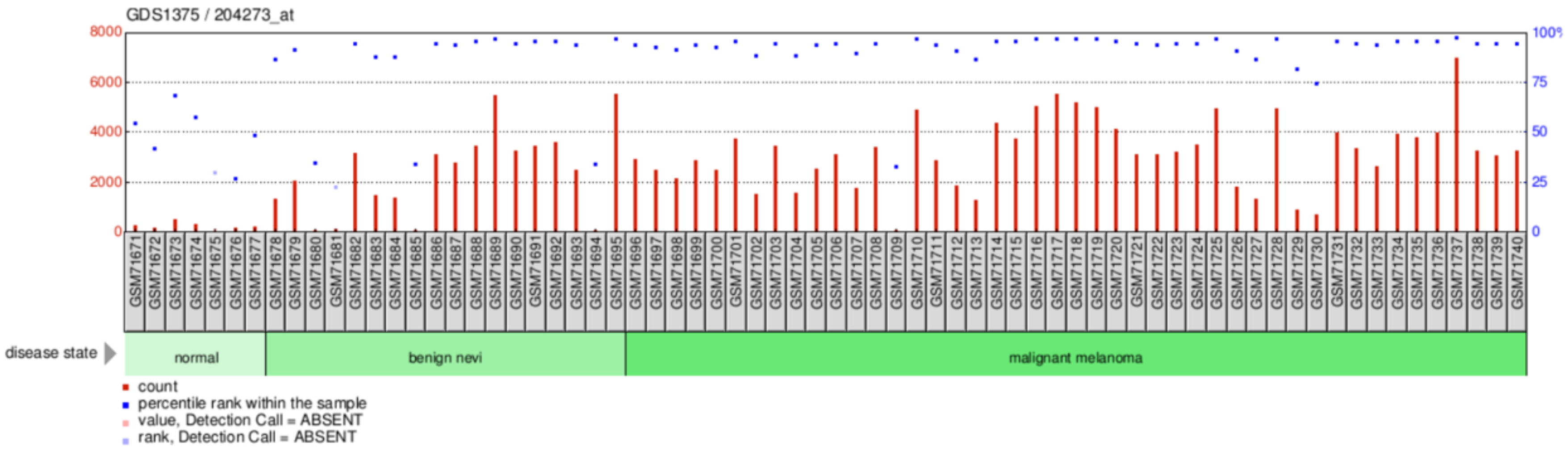

Homo sapiens endothelin receptor type B (EDNRB), transcript variant 1, mRNA
NCBI Reference Sequence: NM_000115.5

Data set a2: Expression profiling array of ET-3 in cutaneous malignant melanoma

EDN3 - Cutaneous malignant melanoma
Annotation: EDN3, endothelin 3
Organism: Homo sapiens
Reporter: GPL96, 208399_s_at (ID_REF), GDS1375, **1908 (Gene ID)**
DataSet type: Expression profiling by array, count, 70 samples
ID: 13267200

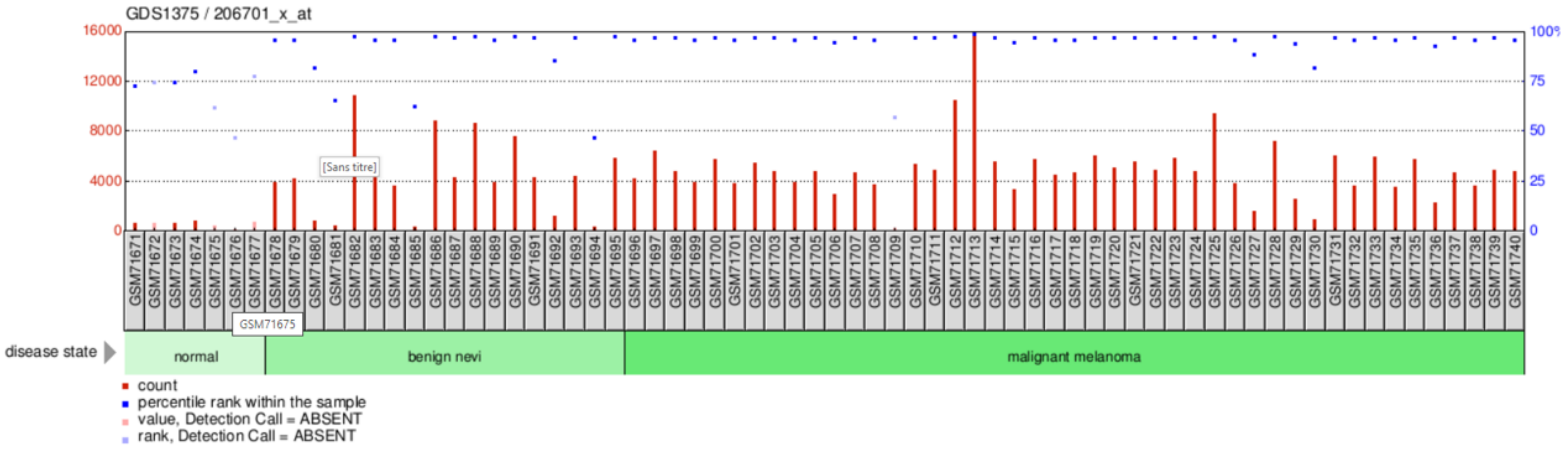

EDN3 endothelin 3 [*Homo sapiens* (human)]
Gene ID: 1908, updated on 5-Mar-2024

Schema a: Schematic representation of binding experiments of RB1 monoclonal antibody in the presence or not of high ET concentration**.**

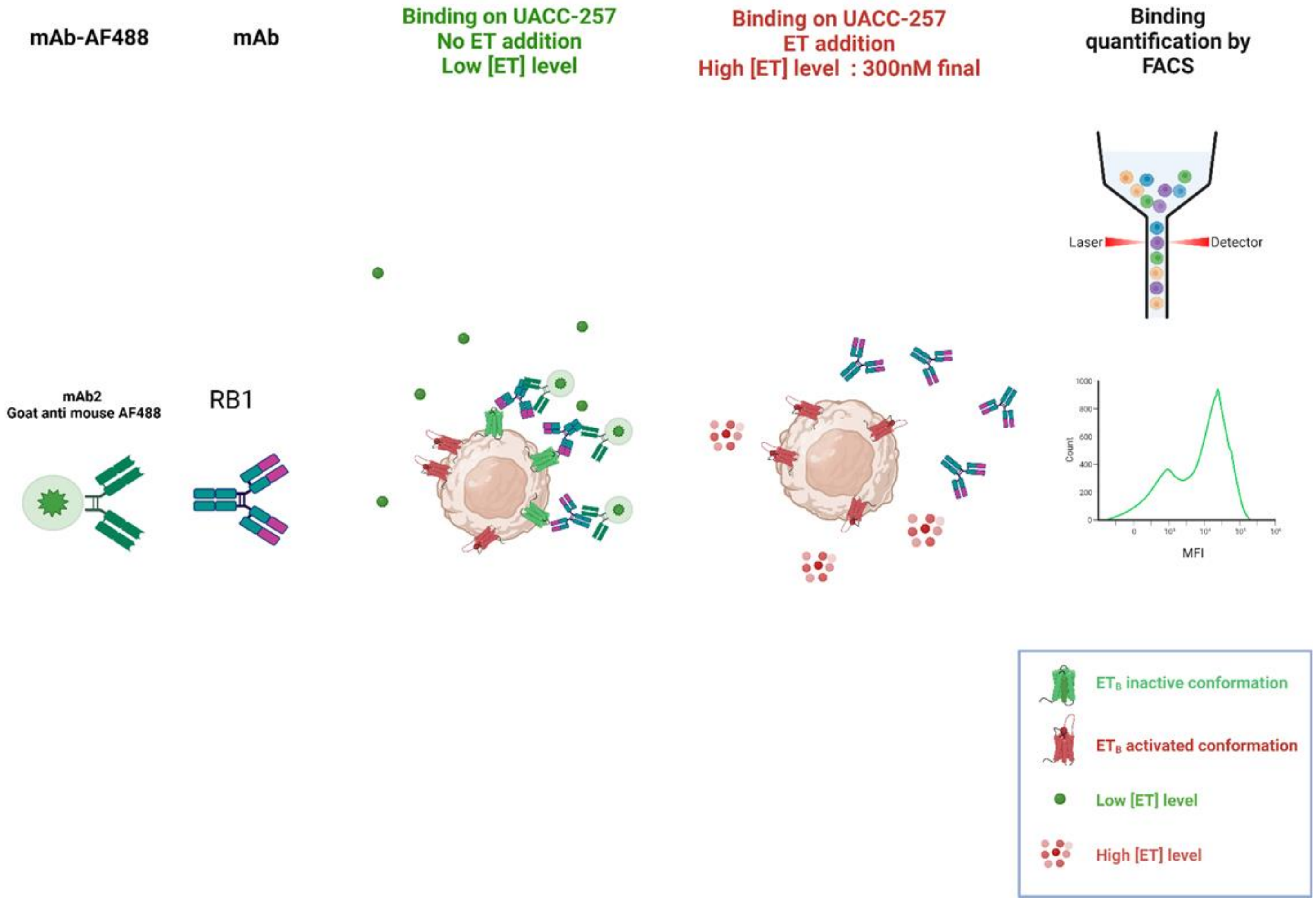

Figure s1

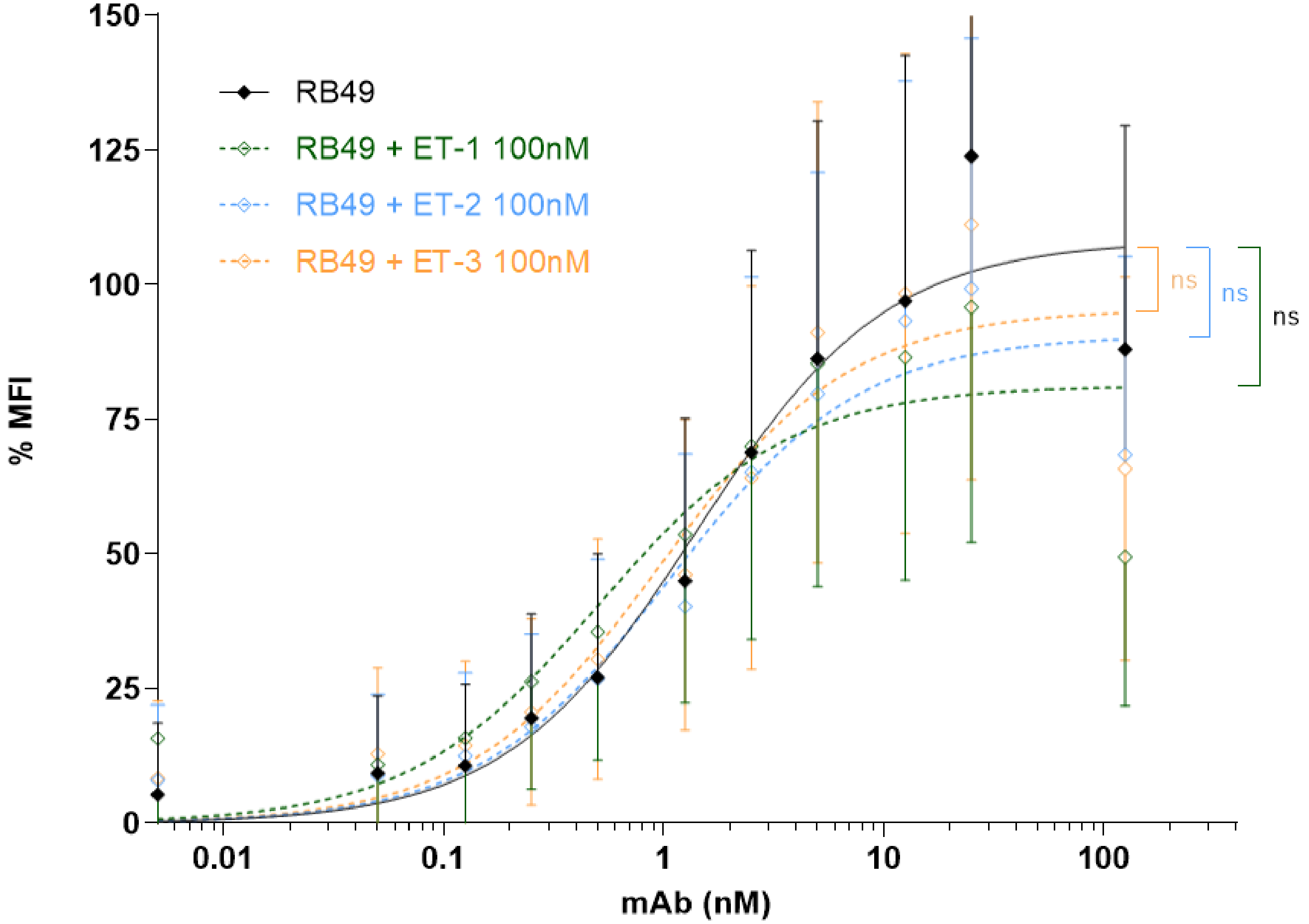

Figure s1 RB49 was tested with three different endothelin types, ET-1, ET-2, and ET-3, on human melanoma cells (UACC-257). We measured mean fluorescence intensity (MFI) for each RB49 concentration using flow cytometry on 10,000 cells. The results were plotted as binding curves, showing %MFI versus RB49 concentration. The solid black line represents RB49 alone, while green, blue, and orange dotted lines represent RB49 with ET-1, ET-2, and ET-3, respectively, each at a concentration of 100 nM. The calculated Kd and Bmax values are detailed in Table S1.

Figure s2

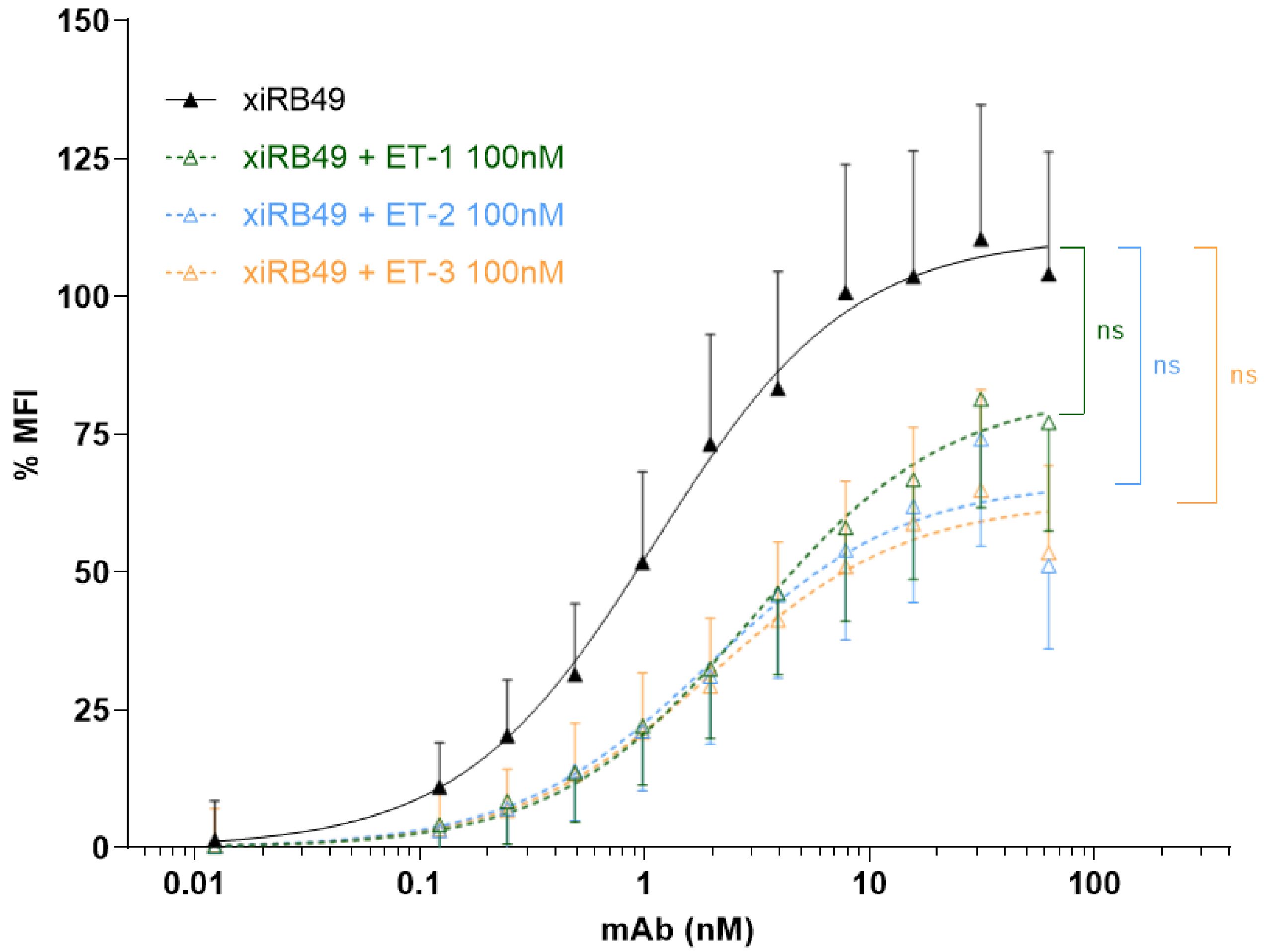

Figure s2 xiRB49 was tested with three different endothelin types, ET-1, ET-2, and ET-3, on human melanoma cells (UACC-257). We measured MFI for each xiRB49 concentration using flow cytometry on 10,000 cells. The results were plotted as binding curves, showing %MFI versus xiRB49 concentration. The solid black line represents xiRB49 alone, while green, blue, and orange dotted lines represent xiRB49 with ET-1, ET-2, and ET-3 respectively, each at a concentration of 100 nM. The calculated Kd and Bmax values are detailed in Table S2.

Figure s3

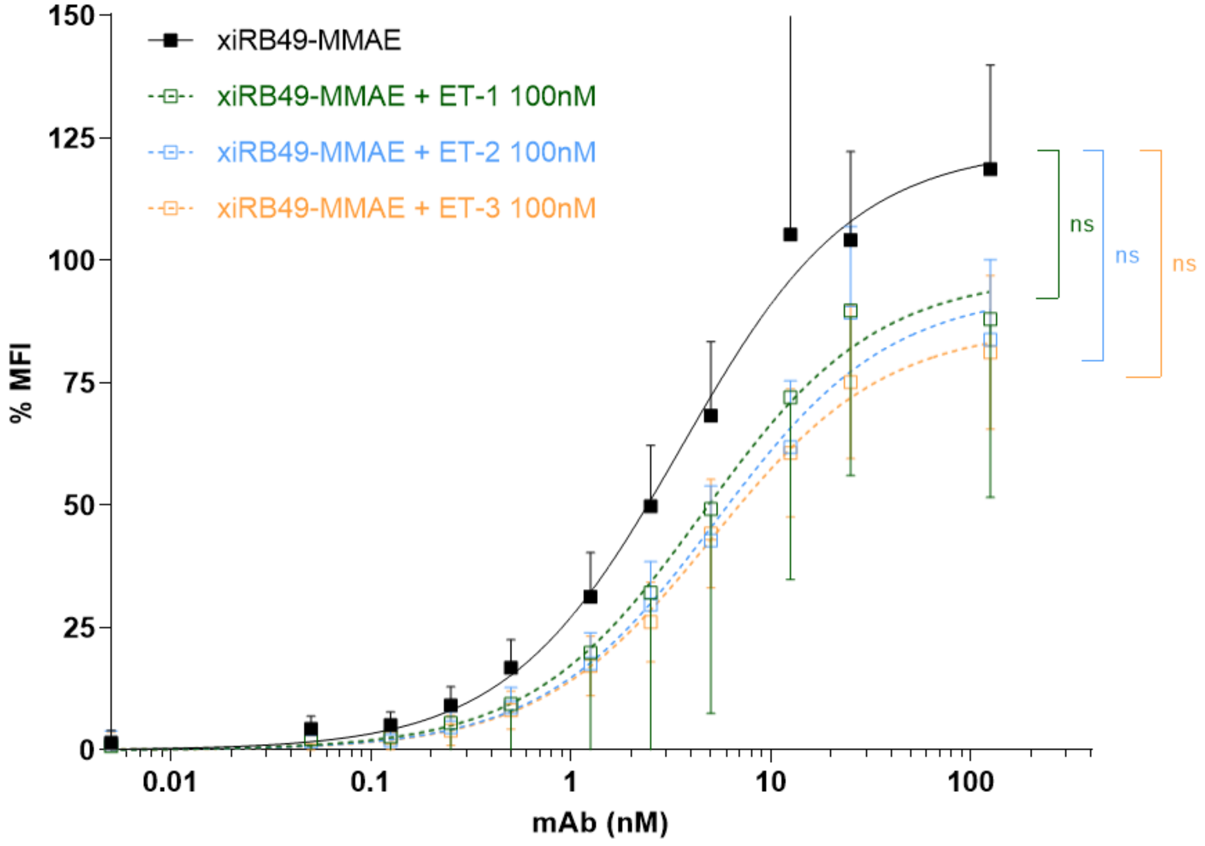

Figure s3 xiRB49-MMAE was tested with three different endothelin types, ET-1, ET-2, and ET-3, on human melanoma cells (UACC-257). We measured MFI for each xiRB49-MMAE concentration using flow cytometry on 10,000 cells. The results were plotted as binding curves, showing %MFI versus xiRB49-MMAE concentration. The solid black line represents xiRB49-MMAE alone, while green, blue, and orange dotted lines represent xiRB49-MMAE with ET-1, ET-2, and ET-3 respectively, each at a concentration of 100 nM. The calculated Kd and Bmax values are detailed in Table S3.

Figure s4

ET$_B$ **ET$_A$**

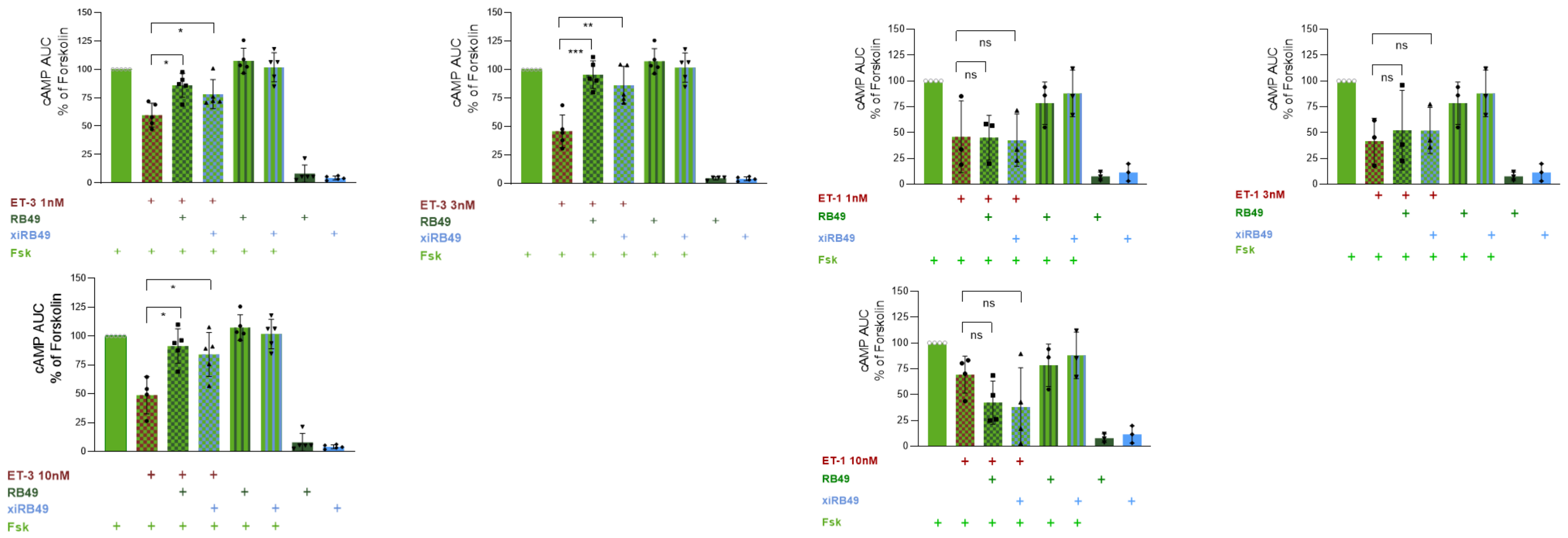

Figure s4 Inhibition of cAMP production induced by forskolin after ET ligand binding to ET$_A$ and ET$_B$. cAMP is inhibited by Gi-coupled ET$_B$ (left) or ET$_A$ (right) stimulated with various concentrations of ET-3 or ET-1, respectively. RB49 or xiRB49 at 100 nM blocks ET$_B$/ET-3, but not ET$_A$/ET-1-mediated cAMP signaling inhibition.

Figure s5

a RB49

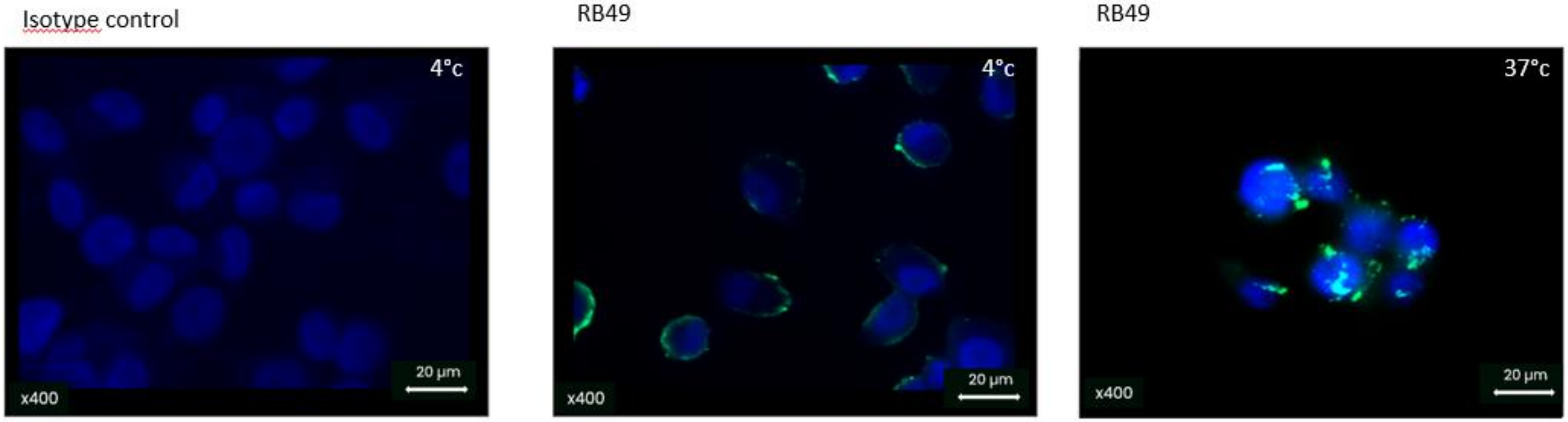

b xiRB49

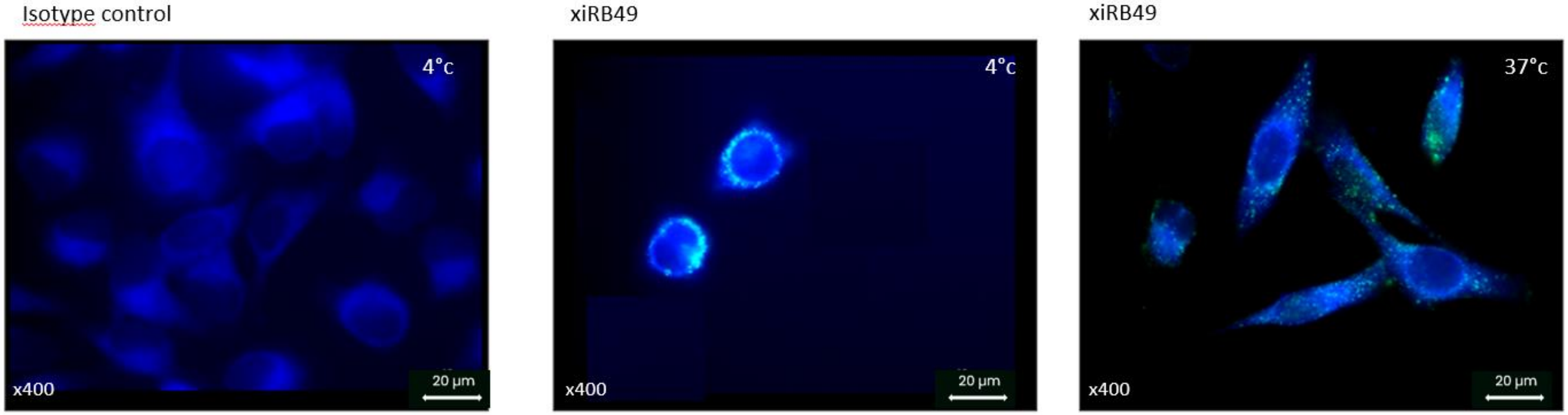

Figure s5 (a) RB49 is internalized at 37°C in UACC-257 cells. With isotype control, no labeling at 488 nm is detected, only blue staining of cell nuclei was observed. At 4°C, only the cell membrane was labeled with RB49 and cell nuclei were stained blue. At 37°C, cytoplasmic fluorescence is mainly observed due to RB49 internalization. The picture is merged with RB49 AF488 fluorochrome labeling and blue staining of cell nuclei at 37°C. (b) xiRB49 is internalized at 37°C in UACC-257 cells. No labeling at 488 nm was detected with the isotype control. Only blue staining of cell nuclei was observed. At 4°C, only the cell membrane was labeled with xiRB49 and cell nuclei were stained blue. At 37°C, cytoplasmic fluorescence is mainly observed due to internalization of xiRB49. The picture is merged with xiRB49 AF488 fluorochrome labeling and blue staining of cell nuclei at 37°C.

Figure s6

a

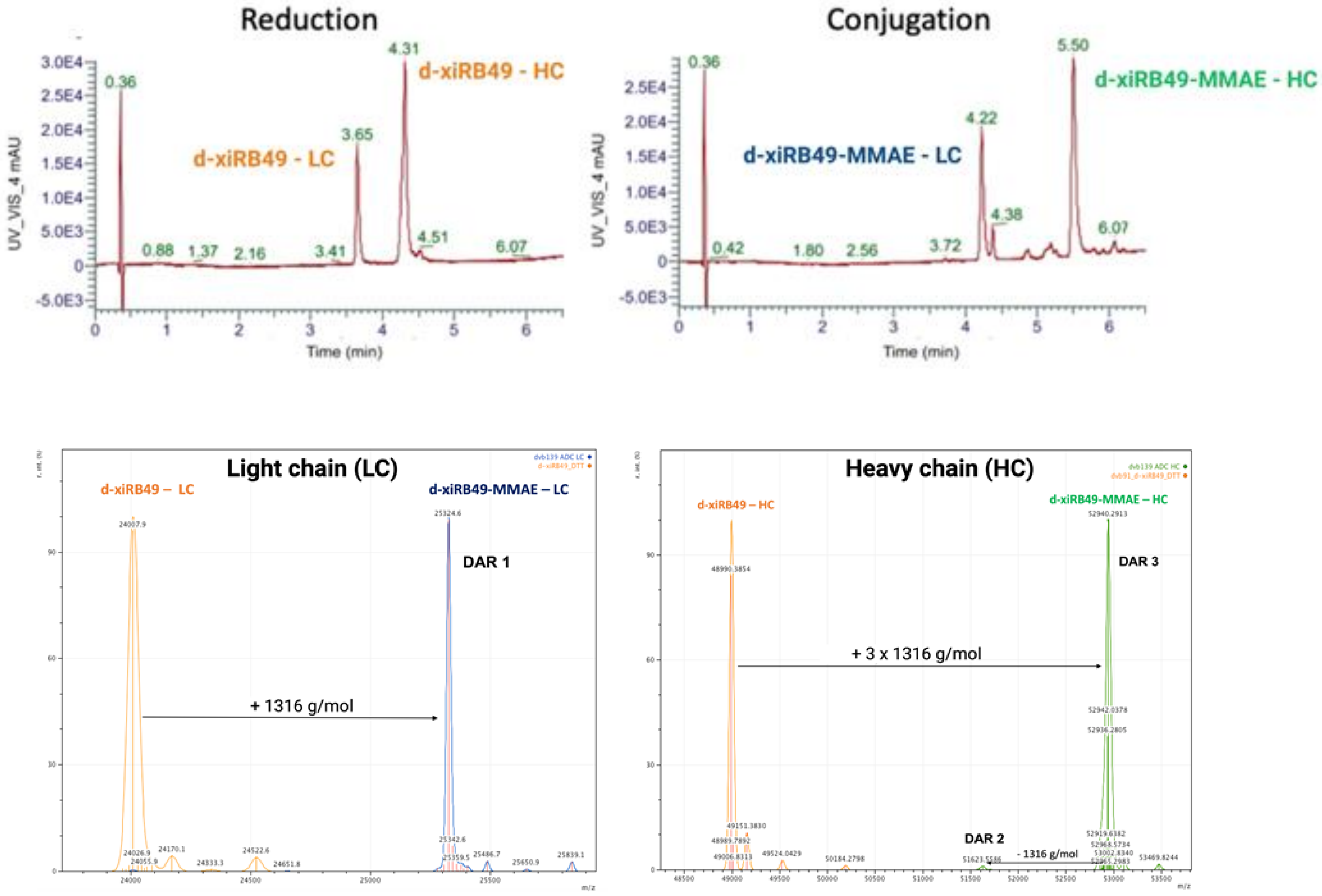

$$DAR_{tot} = (DAR\ LC + DAR\ HC) \times 2 = 8$$

b

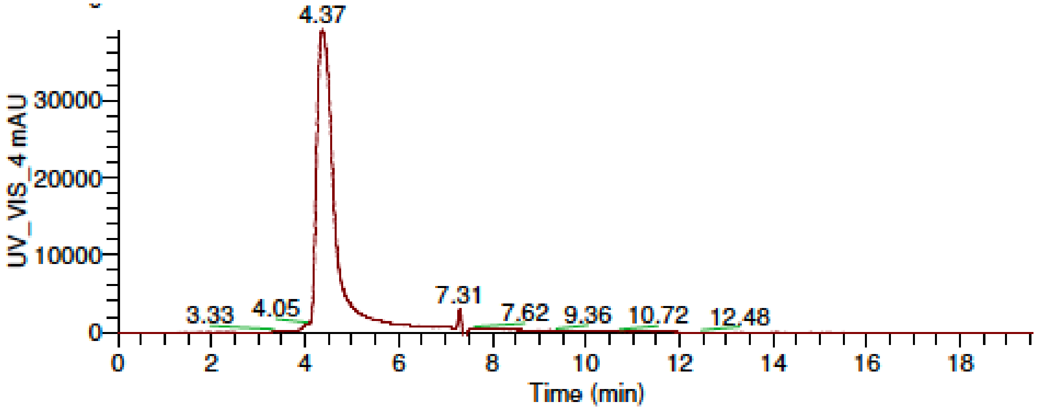
4.37
UV_VIS_4 mAU
30000
20000
10000
0
3.33
4.05
7.31
7.62
9.36
10.72
12.48
0
2
4
6
8
10
12
14
16
18
Time (min)

c

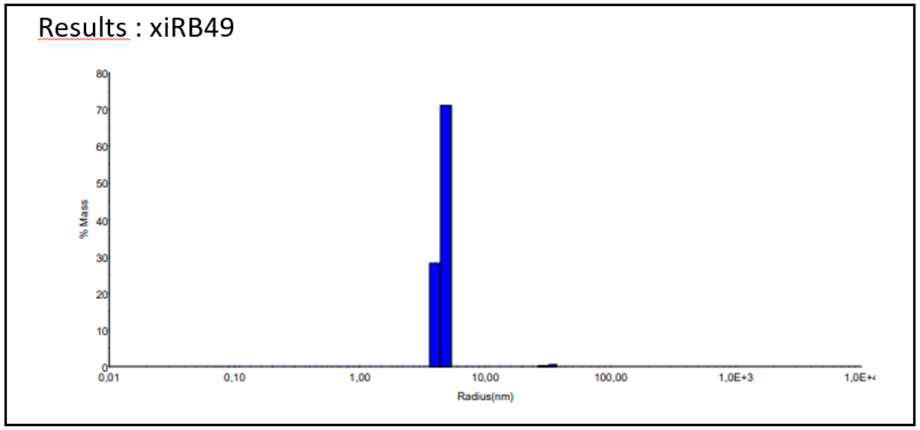
Results : xiRB49
% Mass
Radius(nm)
0,01
0,10
1,00
10,00
100,00
1,0E+3

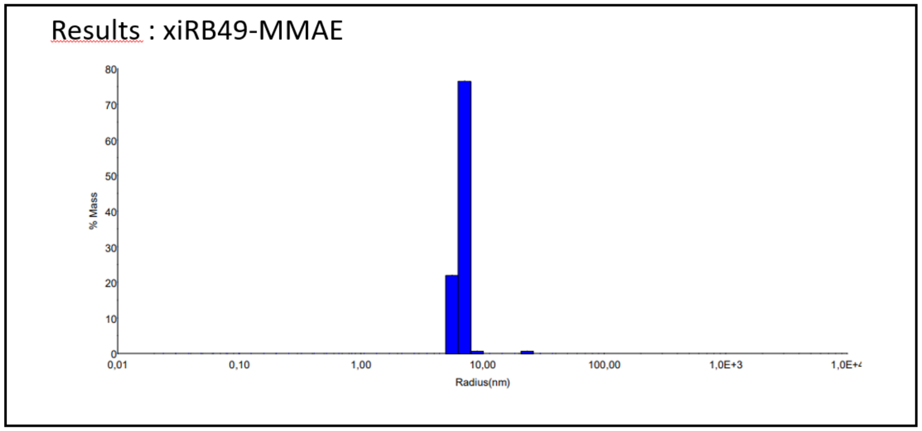
Results : xiRB49-MMAE
% Mass
Radius(nm)
0,01
0,10
1,00
10,00
100,00
1,0E+3

d

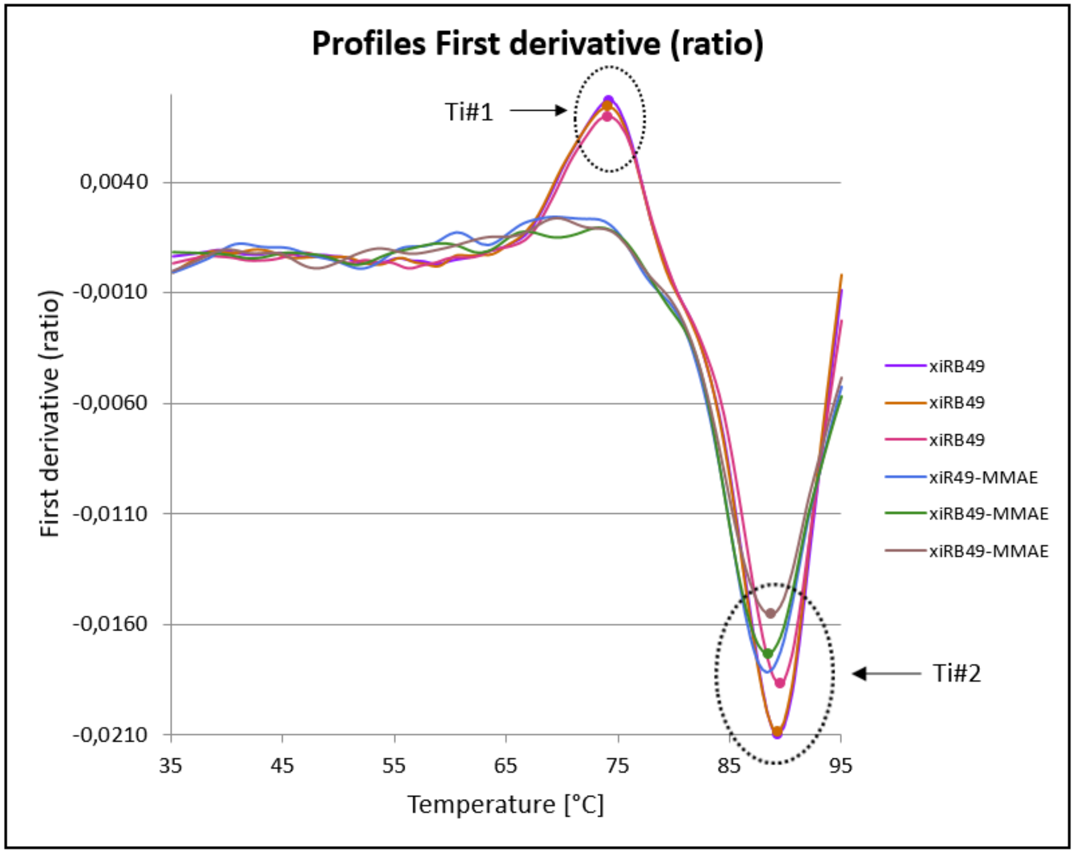

Figure s6 xiRB49-MMAE characterizations. (a) Reduction and conjugation steps of xiRB49-MMAE were followed by reverse-phase HPLC (left: UV chromatogram at 280 nm) coupled to a high-resolution Orbitrap mass spectrometer (right: deconvoluted mass spectra). (b) Aggregation of the final ADC was evaluated by size exclusion chromatography (SEC) with an AdvanceBio SEC column. (c) DLS experiments (n=10) were performed at 25°C and at a final antibody concentration of 0.5 mg/mL in PBS pH 7.4 after centrifugation for 10 minutes at 10,000 g (see Table S6 for results). (d) Thermal denaturation experiments (n=3) were performed in PBS buffer pH 7.4 with an antibody concentration of 0.5 mg/mL (see Table S7 for results).

Figure s7

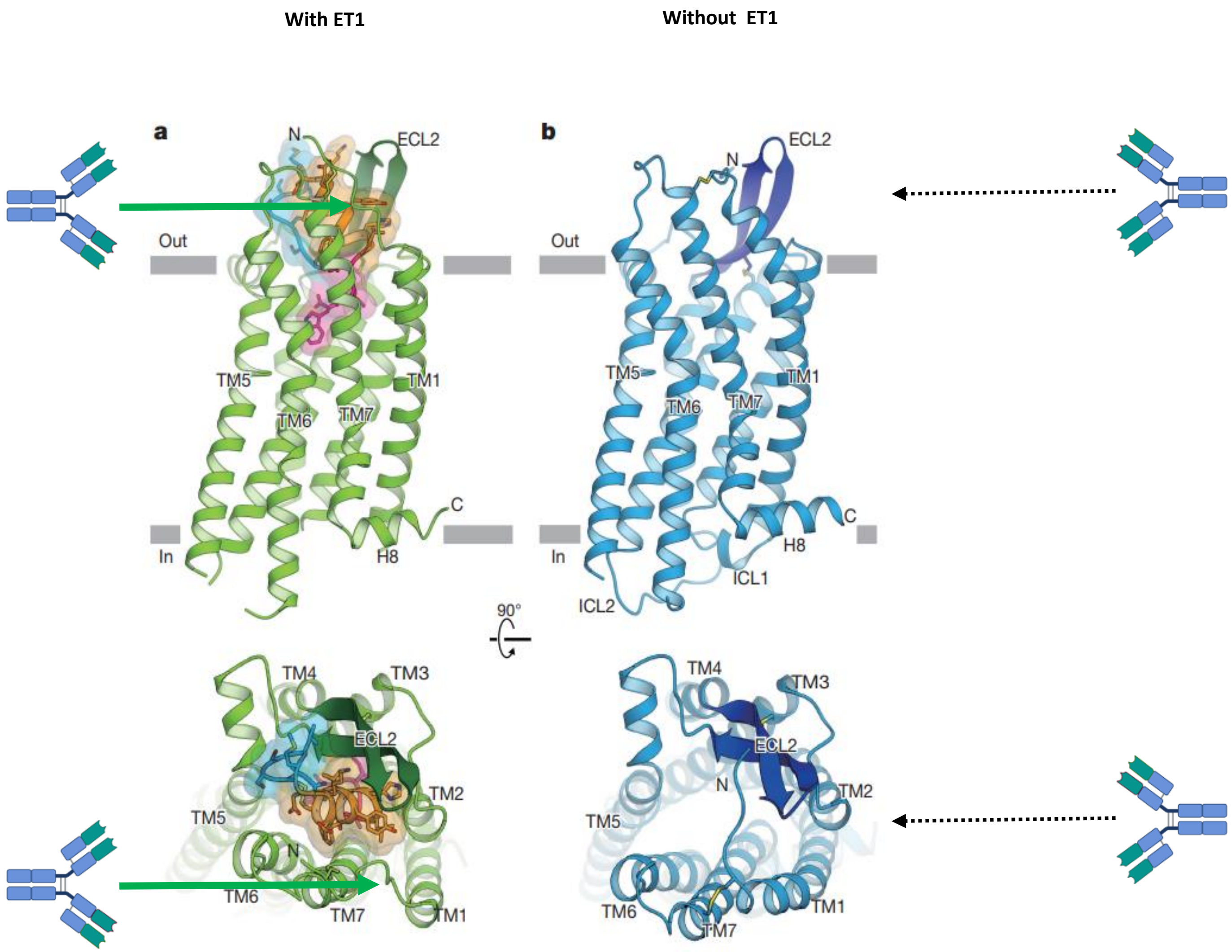

Figure s7 Recognition of the N-terminal domain by xiRB49 of the allosteric ET_B. Adapted from Shihoya, W. *et al.* Activation mechanism of endothelin ET_B receptor by endothelin-1. *Nature* 537, 363 (2016).

Figure s8

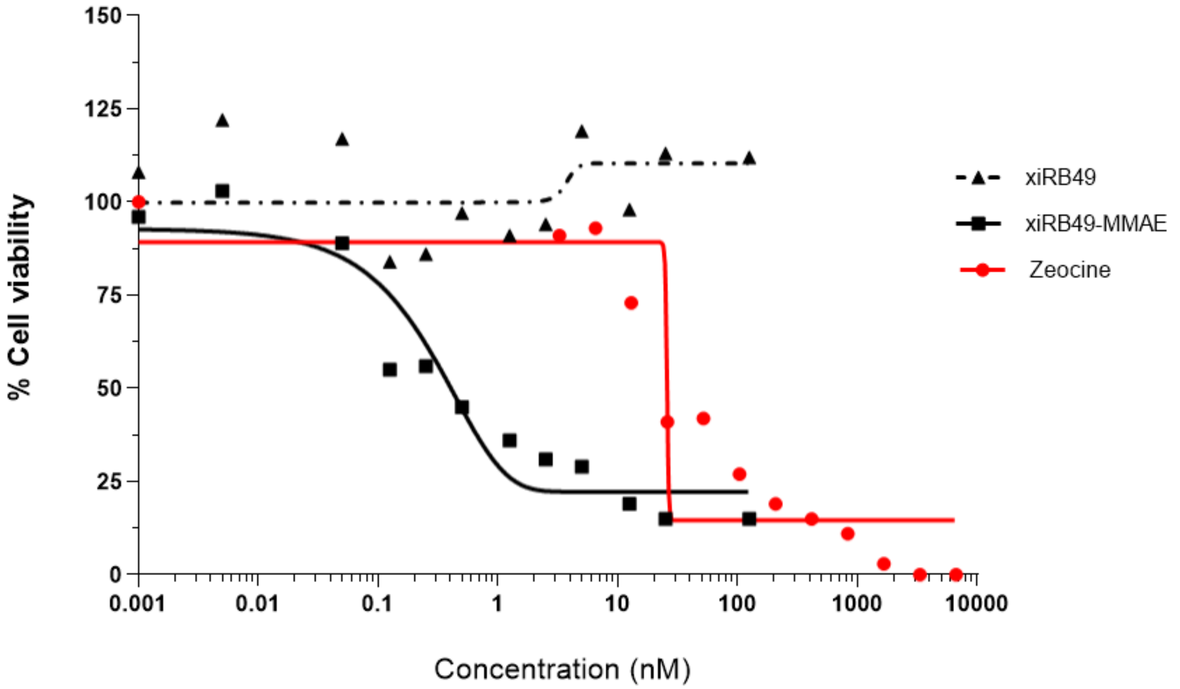

Figure s8 Cytotoxicity experiments were conducted on UACC-257 cells using a specific assay (Abcam, Cambridge, UK- Cat # ref. 112118). The results indicate a significant toxic effect of xiRB49-MMAE, with a calculated IC50 of 0.4 ± 0.1 nM. No toxicity was observed with xiRB49 alone. Zeocin served as a positive control for cytotoxicity with an IC50 of 27.1 ± 7 nM (Table S8).

Tables

Table s1 RB49 binding

Mab	RB49	RB49<br>ET-1 100 nM	RB49<br>ET-2 100 nM	RB49<br>ET-3 100 nM	RB49<br>High ET
**Bmax**	108.1	81.2	90.6	95.5	83.3
**Kd (nM)**	1.4	0.5	1.1	1.0	0.7
**Std. Error**					
Bmax ±	0.5	0.3	0.4	0.4	0.4
Kd ± (nM)	0.02	0.01	0.02	0.02	0.02
**Unpaired T test**					
Significantly different (P < 0.05)?	no	no			
P value summary	ns	ns			
Significantly different (P < 0.05)?	no		no		
P value summary	ns		ns		
Significantly different (P < 0.05)?	no			no	
P value summary	ns			ns	
Significantly different (P < 0.05)?	no				no
P value summary	ns				ns

Table s2 xiRB49 binding

Mab	xiRB49	xiRB49 ET-1 100 nM	xiRB49 ET-2 100 nM	xiRB49 ET-3 100 nM	xiRB49 High ET
**Bmax**<br>**Kd (nM)**	110.7<br>0.8	82.7<br>2.9	66.5<br>1.9	63.0<br>2.0	61.5<br>1.3
**Std. Error**					
Bmax ±<br>Kd ± (nM)	0.2<br>0.01	0.2<br>0.03	0.2<br>0.02	0.2<br>0.02	0.2<br>0.02
**Unpaired T test**					
Significantly different (P < 0.05)?<br>P value summary	no<br>ns	no<br>ns			
Significantly different (P < 0.05)?<br>P value summary	no<br>ns		no<br>ns		
Significantly different (P < 0.05)?<br>P value summary	no<br>ns			no<br>ns	
Significantly different (P < 0.05)?<br>P value summary	no<br>ns				Yes 0.042<br>*

Table s3 xiRB49-MMAE binding

Mab	xiRB49-MMAE	xiRB49-MMAE ET-1 100 nM	xiRB49-MMAE ET-2 100 nM	xiRB49-MMAE ET-3 100 nM	xiRB49-MMAE High ET
**Bmax**	123.2	97.0	93.7	86.5	99.7
**Kd (nM)**	3.5	4.6	5.3	5.1	7.7
**Std. Error**					
Bmax ±	0.5	1.0	0.5	0.5	0.5
Kd ± (nM)	0.05	0.16	0.09	0.10	0.15
**Unpaired T test**					
Significantly different (P < 0.05)?	no	no			
P value summary	ns	ns			
Significantly different (P < 0.05)?	no		no		
P value summary	ns		ns		
Significantly different (P < 0.05)?	no			no	
P value summary	ns			ns	
Significantly different (P < 0.05)?	no				no
P value summary	ns				ns

Table s4 RB1 binding

Mab	RB1	RB1 ET-1 100 nM	RB1 ET-2 100 nM	RB1 ET-3 100 nM	RB1 High ET
**Bmax**	86.0	4.8	9.4	5.3	3.0
**Kd (nM)**	0.9	NA	NA	NA	NA
**Std. Error**					
Bmax ±	0.4	0.8	0.2	0.2	0.2
Kd ± (nM)	0.2	NA	NA	NA	NA
**Unpaired T test**					
Significantly different (P < 0.05)?<br>P value summary	Yes 0.0010<br>**	Yes 0.0010<br>**			
Significantly different (P < 0.05)?<br>P value summary	Yes 0.0015<br>**		Yes 0.0015<br>**		
Significantly different (P < 0.05)?<br>P value summary	Yes 0.0007<br>***			Yes 0.0007<br>***	
Significantly different (P < 0.05)?<br>P value summary	Yes 0.0007<br>***				Yes 0.0007<br>***

NA = Not Applicable

Table s5 Rh determination by DLS

Mab	xiRB49	xiRB49-MMAE
**Radius (nm)**	4.6	6.8
**Std. Error**		
Radius (nm) ±	0.2	0.1

Table s6 Ti determination by thermal denaturation curves

Mab	xiRB49	xiRB49-MMAE
**Ti#1 (°C)**<br>**Ti#2 (°C)**	74.1<br>89.4	-<br>88.5
**Std. Error**		
Ti#1 (°C) ±<br>Ti#2 (°C) ±	0.08<br>0.13	-<br>0.17

Table s7 IC50 determination by viability cell assay

Mab	xiRB49	xiRB49-MMAE	Zeocin
**IC50 (nM)**	NA	0.4	27.1
**Std. Error**			
IC50 (nM) ±	NA	0.1	7.2
